# A missing piece of the puzzle in pulmonary fibrosis: anoikis resistance promotes fibroblast activation

**DOI:** 10.1186/s13578-022-00761-2

**Published:** 2022-02-25

**Authors:** Juan Yin, Jing Wang, Xinxin Zhang, Yan Liao, Wei Luo, Sha Wang, Jiawei Ding, Jie Huang, Mengling Chen, Wei Wang, Shencun Fang, Jie Chao

**Affiliations:** 1 Jiangsu Provincial Key Laboratory of Critical Care Medicine, Zhongda Hospital, Department of Physiology, School of Medicine, Southeast University, #87 Dingjiaqiao Rd, Nanjing, 210009 Jiangsu China; 2 School of Life Science and Technology, Southeast University, Nanjing, 210009 Jiangsu China; 3 Key Laboratory of Environmental Medicine Engineering, Ministry of Education, School of Public Health, Southeast University, Nanjing, 210009 Jiangsu China; 4 Department of Respiratory Medicine, Nanjing Chest Hospital, the Affiliated Brain Hospital of Nanjing Medical University, Nanjing, 210029 Jiangsu China; 5 School of Medicine, Xizang Minzu University, Xianyang, 712082 Shanxi China

**Keywords:** Anoikis, ZC3H4, Pulmonary fibrosis, Fibroblast, ECM

## Abstract

**Background:**

Pulmonary fibrosis initiates a pneumonic cascade that leads to fibroblast dysfunction characterized by excess proliferation. Anoikis is a physiological process that ensures tissue development and homeostasis. Researchers have not clearly determined whether disruption of anoikis is involved in pulmonary fibrosis.

**Results:**

Here, we investigated the mechanism by which silica induces fibroblast activation via anoikis resistance and subsequent fibrosis. Anoikis of lung fibroblasts, alveolar epithelial cells and endothelial cells during the process of fibrosis was detected using CCK-8, western blot, cell count and flow cytometry (FCM) assays. Although the three cell types showed similar increases in proliferation, the expression of NTRK2, a marker of anoikis resistance, was upregulated specifically in fibroblasts, indicating the unique proliferation mechanism of fibroblasts in pulmonary fibrosis, which may be related to anoikis resistance. Furthermore, the CRISPR/Cas9 system was used to investigate the molecular mechanism of anoikis resistance; the SiO_2_-induced inflammatory response activated the MAPK/PI3K signaling pathway in lung fibroblasts and then induced the expression of the ZC3H4 protein, which specifically mediated anoikis resistance, followed by pulmonary fibrosis.

**Conclusions:**

The current study revealed a specific pattern of fibroblast proliferation, and strategies targeting anoikis resistance may inhibit the pathological process of pulmonary fibrosis. This result provides a new approach for treating pulmonary fibrosis and new insights into the potential application of ZC3H4 in the development of novel therapeutic strategies for mitigating pulmonary fibrosis.

**Supplementary Information:**

The online version contains supplementary material available at 10.1186/s13578-022-00761-2.

## Introduction

Pulmonary fibrosis is a lung disease caused by abnormal wound healing in susceptible people with repeated alveolar injury. This condition mainly manifests as chronic inflammation and collagen deposition in the extracellular matrix (ECM), which eventually leads to diminished lung function and is the final step in the development of many lung diseases [1, 2]. This process is characterized by continuous and irreversible damage and involves a variety of lung cells, such as fibroblasts [3], epithelial cells [4], and endothelial cells. To date, the mechanism of pulmonary fibrosis has not been completely elucidated. The excess proliferation of lung cells during pulmonary fibrosis is similar to the proliferation of detached cells caused by anoikis resistance during tumor invasion or migration and skin injury [5]. Anoikis, which was first identified in 1993, is a unique programmed death phenomenon caused by adherent cells leaving the ECM and is a special form of cell apoptosis. Anoikis occurs by preventing the reattachment of exfoliated cells to a new matrix, preventing improper aggregation and growth. This process is closely related to growth and development, tissue homeostasis, and cancer metastasis [6]. The ECM is a complex multimolecular protein network that provides structural support for cells and serves as an adherent substrate for cell migration [7]. During the processes of angiogenesis and wound healing, reshaping of the ECM is essential and is regarded as a physiological process to ensure appropriate tissue development and homeostasis [7]. The dynamic balance between cells and the surrounding microenvironment is maintained by preventing detached cells from reattaching to a new matrix and growing in the wrong place [8]. Thus, failure to execute the anoikis program may result in detached cells surviving in a suspension or proliferating at ectopic sites in the ECM that differ from the primary matrix. Removal of the restriction on anoikis execution was identified as a hallmark of malignancy and facilitates the survival of cancer cells in the circulatory system to complete their metastasis to distant organs [9, 10]. According to previous studies, the zinc finger protein MCP-1-inducible protein (MCPIP1) and the newly discovered zinc finger protein ZC3H4 are involved in fibrosis [11]. MCPIP1 (also known as ZC3H12A) is involved in silica-induced pulmonary fibrosis and regulates macrophage and fibroblast activation [12]. ZC3H4 and MCPIP1 belong to the CCCH-type zinc finger protein family. In RAW264.7 mouse macrophages, ZC3H4 promoted the activation of macrophages and downstream fibroblasts [13]. In the current study, the zinc finger protein ZC3H4 was shown to promote anoikis resistance in fibroblasts and subsequently induce pulmonary fibrosis. These findings reveal a novel role for anoikis in silica-induced pulmonary inflammation and suggest that ZC3H4 is a target for the clinical diagnosis and treatment of pulmonary fibrosis.

## Materials and methods

### Reagents

Silicon dioxide, in which 80% of particles exhibited a diameter of less than 5 μm, was purchased from Sigma® (S5631), processed via sedimentation according to Stokes’ law, subjected to acid hydrolysis, and heated overnight (200 °C for 16 h) [14]. Antibodies against ZC3H4 (2004 1–1-AP, rabbit), TrkB (13129-1-AP, rabbit), BIP (11578-1-AP, rabbit), CHOP (15204-1-AP, rabbit), ATF6α (66563-1-AP, mouse), ATG5 (60061-1-AP, mouse), BECN1 (11306-1-AP, rabbit), and LC3B (14600-1-AP, rabbit) were purchased from Proteintech, Inc. Antibodies against Akt (4691S, rabbit), p-Akt (4060S, rabbit), JNK (9258S, rabbit), p-JNK (4668S, rabbit) and IRE1α (3294, rabbit) were purchased from CST, Inc. Antibodies against GAPDH (sc-25778, rabbit), BIM (sc-374358) and NPNT (393033, rabbit) were purchased from Santa Cruz Biotechnology.

### Cell culture

Human bronchial epithelial cells (BEAS-2B), human pulmonary microvascular endothelial cells (HPMECs) and human peripheral blood mononuclear cells (THP-1 cells) were purchased from ATCC®. Human pulmonary fibroblasts from adults (HPF-a), mouse pulmonary fibroblasts (MLG) and RAW264.7 cells were purchased from ScienCell and maintained in DMEM containing 10% FBS with 5% CO_2_ and 37 °C in an incubator (Thermo Heracell 150i CO_2_ incubator, Thermo Fisher Scientific, Inc., Germany).

### Establishment of a mouse model of silicosis

The mouse model of silicosis was established as previously described [15]. All experiments were approved by the Institutional Animal Care and Use Committee of the Medical School of Southeast University.

### Spatial transcriptomics (GSE183683)

#### Sample collection

The inclusion criteria for the model group were the same as those for single-cell sequencing. Lung tissues were sliced appropriately in the horizontal direction and then frozen in OCT on dry ice as quickly as possible.

#### Staining and imaging

Cryosections were sliced at a thickness of 10 μm and mounted onto GEX arrays. They were then placed on a Thermocycler Adaptor with the active surface facing up and incubated for 1 min at 37 °C. Afterward, the sections were fixed with methyl alcohol at -20 °C for 30 min and subjected to staining with H&E (Eosin, Dako CS701, Hematoxylin Dako S3309, bluing buffer CS702). Brightfield images were captured using a Leica DMI8 whole-slide scanner at 10 × resolution.

#### Permeabilization and reverse transcription

Spatial gene expression was analyzed using a Visium spatial gene expression slide and reagent kit (10 × Genomics, PN-1000184). For each well, a slide cassette was used to create leakproof wells that would facilitate the addition of reagents. First, 70 μl of permeabilization enzyme were added and incubated with the sections at 37 °C. For NS-7 d, SiO_2_-7 d and NS-56 d, the incubation time was 24 min; for SiO2-56 d, the incubation was 30 min to induce severe lung fibrosis. Each well was washed with 100 μl of SSC, and 75 μl of RT master mix were added to induce cDNA synthesis.

#### Preparation of the cDNA library for sequencing

Upon the completion of first-strand synthesis, the RT master mix was removed from the wells and replaced with 75 μl of 0.08 M KOH. After an incubation for 5 min at room temperature, KOH was aspirated from the wells, and the slices were washed with 100 µl of EB buffer before 75 μl of Second Strand Mix were added to each well for second-strand synthesis. cDNA amplification was performed on a S1000™ Touch Thermal Cycler (Bio-Rad). Visium spatial libraries were constructed using the Visium spatial library construction kit (10 × Genomics, PN-1000184) according to the manufacturer’s instructions. The final libraries were sequenced using an Illumina NovaSeq 6000 sequencer with a sequencing depth of at least 100,000 reads per spot and a paired-end 150 bp (PE150) reading strategy (performed by CapitalBio Technology, Beijing).

### Proteomic analysis of the ECM (PXD028194)

#### Experimental procedures

Total protein was extracted from 6 lung ECM samples (3 for the NS-56 d group, namely, con111, con116, con117; 3 for the SiO_2_-56 d group, namely, M80, M101, M107), and a portion (10 µl) of the protein was used to measure the protein concentration, followed by separation on SDS–PAGE gels. Another part was collected for trypsin digestion and labeled with TMT (tandem mass tag) reagents, con111 with 126, con 116 with 127, con117 with 128, M80 with 129, M101 with 130, and M107 with 131. Equal amounts of each labeled sample were mixed, and an appropriate quantity of protein subjected to chromatographic separation. Finally, the samples were analyzed using LC–MS (liquid chromatography–mass spectrometry).

#### Analysis of LC–MS/MS data

The raw LC–MS/MS data were processed using Proteome Discover 2.4 (Thermo, USA). Based on ≥ 1 unique peptides, any group of samples with an expression level ≥ 50% of the protein was retained. Then, the missing values were imputed with the mean protein expression in the corresponding group. Next, the data were median-normalized and log2-transformed to obtain credible proteins. Afterward, we performed statistical analyses and created a visual display of these proteins using R software (version 4.2) ggplot2 package (version 3.2.2), including principal component analysis (PCA), sample correlation analysis, sample hierarchical clustering analysis, visual display of data after standardization and a density plot.

Using credible proteins, we performed Student’s t test to identify significant differences in proteins in the NS-56 d group and SiO_2_-56 d group. The fold change (FC) was calculated to evaluate the difference in the expression level of a certain protein between samples. The p value (P) calculated using the t test shows the significance of the difference between samples with FC ≥ 2.0 and P ≤ 0.05. The clustering heatmap based on the R software (version 4.2) pheatmap package (version 1.0.12) was used for quality control of standardized experimental data and for displaying the results after the enrichment of differentially expressed proteins. Generally, samples from the same group appear in the same cluster after clustering.

For the identified proteins, annotation information was extracted from the UniProt database. After obtaining the differentially expressed proteins (FC ≥ 2, P ≤ 0.05), GO and KEGG functional enrichment analyses of upregulated proteins were performed with the R software (version 4.2) ggplot2 package (version 3.2.2).

### Bioinfomatics analysis using the Space Ranger pipeline

Space Ranger software was obtained from the 10 × Genomics website (https://support.10xgenomics.com/spatial-gene-expression/software/downloads/latest). Alignment, filtering, barcode counting, and UMI counting were performed with the Space Ranger Count module to generate a feature-barcode matrix and determine clusters. Dimensionality reduction was performed using principal component analysis (PCA), and the first ten principal components were used to generate clusters with the K-means algorithm and graph-based algorithm.

### CRISPR/Cas9 plasmid transfection

Fibroblasts were transiently transfected with CRISPR/Cas9 plasmids according to the manufacturer’s recommended protocol (Santa Cruz®) to delete or upregulate ZC3H4 expression and examine the downstream effects. The transfection efficiency was determined via western blotting. Briefly, HPF-a cells were seeded in a 6-well plate at a density of 2 × 10^5^ cells per well in 3 mL of antibiotic-free standard growth medium and grown to 40–80% confluence. Then, 300 µL of the plasmid DNA/UltraCruz® Transfection Reagent Complex, consisting of 2 µg of plasmid DNA and 10 µL of the UltraCruz® Transfection Reagent in Plasmid Transfection Medium, were added dropwise to each well. Thereafter, gentle mixing was performed by swirling the plate, and the cells were incubated for 24–72 h under normal conditions prior to their use in subsequent experiments.

### Anoikis model

Poly-2-hydroxyethyl methacrylate (poly-HEMA, Sigma, USA) was prepared in anhydrous ethanol at a concentration of 10 mg/mL. Next, 1.5 mL of the poly-HEMA solution was added to a 6-well plate and then dried for 24 h under sterile conditions. The wells coated with poly-HEMA were immediately exposed to ultraviolet light for disinfection and washed twice with sterile PBS. Approximately 5 × 10^5^ cells in a single-cell suspension were added to each well and cultured at 37 °C with 5% CO2 for 48 h. The cells were then collected and washed twice with sterile PBS. Flow cytometry (FCM) was used to detect the incidence of anoikis among cells stained with an Annexin V-PE apoptosis assay kit (KeyGEN BioTECH, KGA108, Nanjing, China).

### Extracellular matrix model of mouse lung tissue

Tissues were embedded in OCT compound, frozen and sliced into 200 μm sections with a cryotome before they were placed into tissue culture dishes containing 15 mL of lysis solution (prepared with 1% SDS in deionized water) for digestion. The solution was then changed to 1% Triton X-100 in deionized water. After the samples were washed with PBS, DNase (20 μg/mL) and MgCl (4.2 mM) were added to the tissue to remove protein. Tissues were sterilized with 0.18% peroxyacetic acid and 4.8% acetic acid for 20 min, washed with sterile PBS three times, and stored at 4 °C. Mouse tissues that were approximately the same size were selected and fixed to the bottoms of a 24-well culture plate. HPF-a cells were seeded on top of the tissues at a density of 1 × 10^6^ cells and incubated at 37 °C for 1 h. When the cells adhered to the tissues, 1 mL of complete medium was prepared. The tissue gel was washed twice with PBS before it was digested with collagenase (5 mg/mL), and the sample was cultured for 2–4 days and then collected for cell counting.

### Statistics

The data are presented as the means ± standard errors of the means (SEM). Statistical analyses were performed using Student’s t test or two-way analysis of variance (ANOVA) with GraphPad 8.0 software. The tests used are indicated in the figure legends. The ANOVA results were considered significant at p < 0.05.

## Results

### ***SiO***_***2***_***-induced pulmonary inflammation caused anoikis resistance specifically in fibroblasts***

**Fig. 1:**
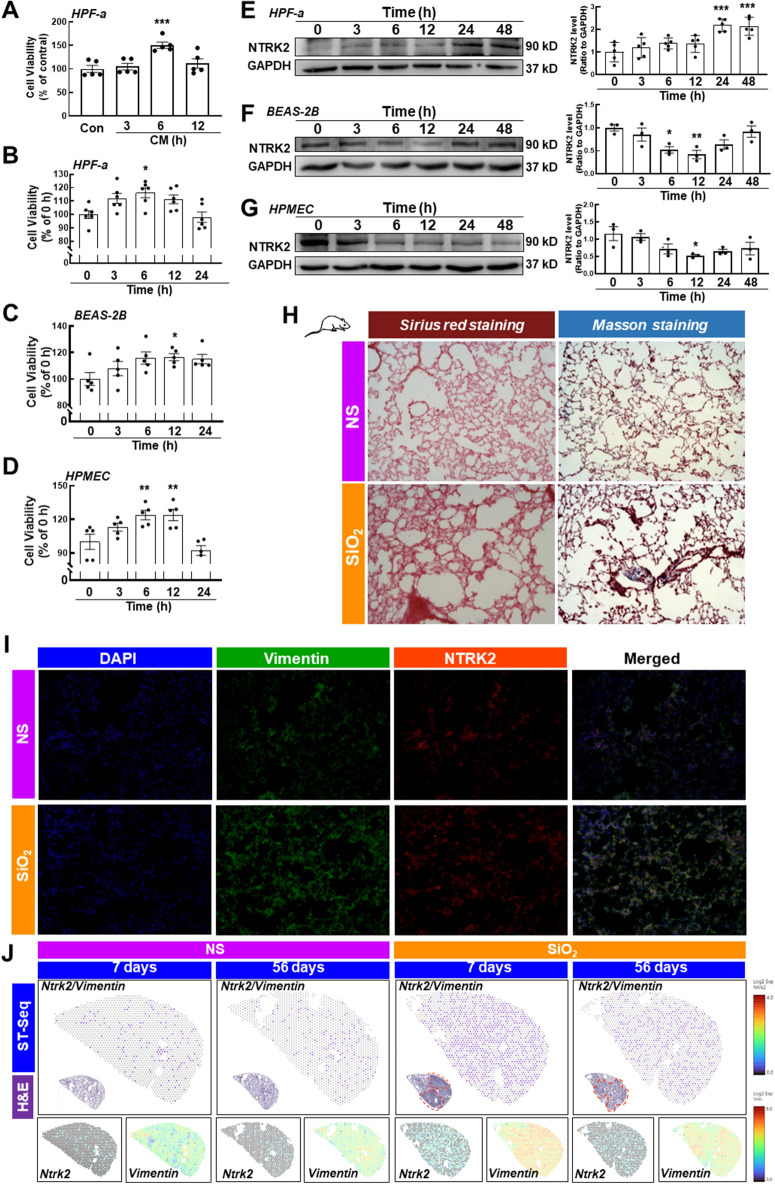
SiO_2_-induced pulmonary inflammation induced anoikis resistance only in HPF-a cells. **A** The CCK-8 assay suggested that a 6-h CM treatment significantly increased the viability of HPF-a cells (n = 5); *P < 0.05 compared with the control group. CM from macrophages treated with SiO_2_ increased the viability of HPF-a cells (**B**), BEAS-2B cells (**C**) and HPMECs (**D**) (n = 5); *P < 0.05 compared with the control group. Representative western blot and densitometry analyses of five separate experiments showing the effects of CM on the expression of the anoikis resistance marker NTRK2 in HPF-a (**E**), BEAS-2B (**F**) and HPMECs (**G**); (n = 5); *P < 0.05 compared with the 0-h group, **P < 0.01 compared with the 0-h group, and ***P < 0.001 compared with the 0-h group. **H** Sirius Red staining and Masson’s trichrome staining showed that SiO_2_ induced more collagen deposition and incomplete pulmonary alveoli in the lung tissues, indicating that the mouse silicosis model was successfully established. Scale bar = 100 μm. **I** Immunofluorescence staining shows the expression of the fibroblast marker Vimentin and the anoikis protein NTRK2 in mouse lung tissue. Scale bar = 100 μm. (J) Spatial transcriptomics showing the coexpression of *Ntrk2* and *Vimentin*

The conditioned medium (CM) of alveolar macrophages was collected, including SiO_2_-treated macrophage CM and non-SiO_2_-treated macrophage CM, and then cocultured with lung cells to simulate the silica-stimulated inflammatory environment in the lung. Based on the SiO_2_ dose–response relationship experiment conducted in an previous study [13], we set the SiO_2_ concentration to 50 μg/cm^2^. HPF-a cells were selected to screen the appropriate CM, and CM from macrophages exposed to SiO_2_ for 6 h induced a significant increase in HPF-a cell viability; thus, this CM was selected for all subsequent experiments (Fig. 1A). On the other hand, to exclude the effect of macrophages, we also used these two types of CM cocultured with the three types of lung cells. After 6 h of coculture, SiO_2_-treated macrophage CM increased the cellular activity of the three cell lines when compared with non-SiO_2_-treated macrophage CM. In addition, the TrkB protein level was increased and BIM was decreased in HPF-a cells after 24 h of coculture (Additional file 1: Figure S1B–E). Then, lung fibroblasts, epithelial cells, and endothelial cells were incubated with CM. As shown in Fig. 1B–D, CM treatment increased the activity of all three types of cells (Fig. 1B–D), while only fibroblasts exhibited increased expression of the NTRK2 (TrkB, a molecular marker of anoikis resistance) protein, which peaked at 24 and 48 h after CM stimulation (Fig. 1E–G). We established a mouse model of silicosis to validate the in vitro findings. Sirius Red staining and Masson’s trichrome staining showed substantial damage to the alveolar cavity and increased collagen deposition in the SiO_2_ group, indicating the successful establishment of pulmonary fibrosis (Fig. 1H). The statistical analysis of the fibrotic area detected using Masson’s trichrome staining is shown in Additional file 1: Figure S1A. Immunofluorescence staining showed that the expression levels of the fibroblast markers vimentin, α-SMA and collagen 1 in lung tissue were increased in the SiO_2_ group compared with the normal saline (NS) group, confirming the occurrence of pulmonary fibrosis (Fig. 1I and Additional file 1: Figure S1F, G). Consistent with the in vitro experiments, the expression of NTRK2 in the lung tissue of the SiO_2_ group was increased and colocalized with vimentin (Fig. 1I). In contrast, a similar pattern was not observed in lung epithelial cells or pulmonary endothelial cells (Additional file 1: Figure S1H, I), suggesting that anoikis resistance uniquely occurred in fibroblasts after CM stimulation. Furthermore, spatial transcriptomics results suggested that *Ntrk2* expression was specifically increased in fibroblasts, as indicated by the colocalization of *Ntrk2* and *Vimentin* (Fig. 1J). Ntrk2 also colocalized with Acta2 (Additional file 1: Figure S1J), confirming anoikis resistance in fibroblasts.

### Proliferation, but not apoptosis, contributed to anoikis resistance in fibroblasts

**Fig. 2:**
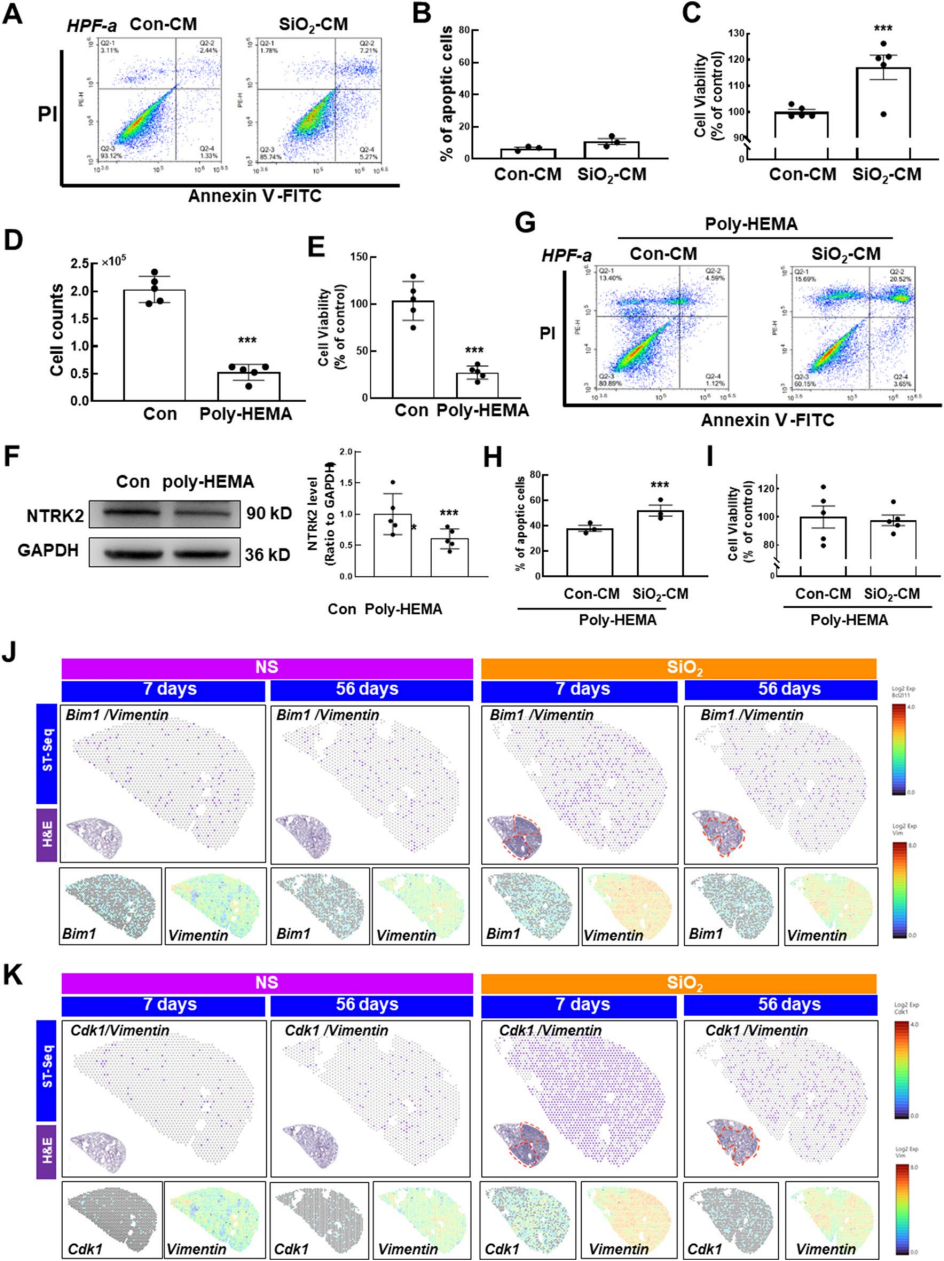
Anoikis resistance-mediated fibroblast activation in response to SiO_2_. **A** HPF-a cells were treated with Con-CM and SiO_2_-CM for 24 h and double stained with Annexin V and PI. The sum of the counts in Q2-1, Q2-2 and Q2-4 was defined as the number of apoptotic cells. **B** Statistical analysis of three independent experiments of HPF-a cell apoptosis using FCM. **C** The CCK-8 assay detected the viability of HPF-a cells after treatment with Con-CM and SiO_2_-CM for 24 h (n = 5). ***P < 0.001 compared with the control group. **D** HPF-a cells were cultured in wells pretreated with poly-HEMA for 48 h and collected for counting (n = 5). ***P < 0.001 compared with the control group. **E** HPF-a cells were suspended for 48 h, and cell viability was detected using the CCK-8 assay (n = 5). ***P < 0.001 compared with the control group. **F** HPF-a cells were suspended for 48 h, and the expression level of TrkB was detected (n = 5). ***P < 0.001 compared with the control group. **G** HPF-a cells were suspended for 48 h before they were treated with Con-CM and SiO_2_-CM for 24 h and double stained with Annexin V and PI. The sum of the counts in Q2-1, Q2-2 and Q2-4 was defined as the number of apoptotic cells. **H** Statistical analysis of three independent experiments of HPF-a cell apoptosis using FCM; ***P < 0.001 compared with the attached group. **I** Statistical analysis of five independent experiments of HPF-a cell viability using the CCK-8 assay (n = 5); ***P < 0.001 compared with the attached group. **J** Spatial transcriptomics showed the coexpression of apoptosis markers (*Bim1)* and *Vimentin*. **K** Spatial transcriptomics showing the coexpression of proliferation markers (*Cdk1*) and *Vimentin*

Anoikis is a type of apoptosis that occurs in response to inappropriate cell/ECM interactions, indicating a special form of cell death initiated by signals not used in response to other proapoptotic insults. We used FCM to further elucidate the process of anoikis resistance induced by SiO_2_-CM. To our surprise, SiO_2_-CM tended to slightly increase apoptosis in fibroblasts (Fig. 2A, B) but induced a significant increase in cell viability (Fig. 2C), indicating that the anoikis resistance induced by SiO_2_-CM may not be due to a decrease in apoptosis. We used poly-HEMA to establish an in vitro anoikis model and confirm the role of anoikis resistance in proliferation induced by SiO_2_-CM. Upon culture in the presence of poly-HEMA, the cells no longer adhered to the plate but showed a state of agglomeration and suspension growth (Additional file 1: Figure S2A). After 48 h, the cells exhibited obvious apoptosis, and the number of cells and the vitality of cells seeded in poly-HEMA-precoated plates were significantly reduced compared to those seeded in uncoated plates (Fig. 2D, E). In addition, the TrkB protein level was also reduced when HPF-a cells were cultured in poly-HEMA plates (Fig. 2F). Interestingly, the SiO_2_-CM-induced slight increase in apoptosis was augmented by poly-HEMA pretreatment (Fig. 2G, H), excluding the role of apoptosis in anoikis resistance. Furthermore, poly-HEMA pretreatment abolished the increase in the viability of fibroblasts induced by SiO_2_-CM, confirming the role of cell proliferation in anoikis resistance.

**Fig. 3:**
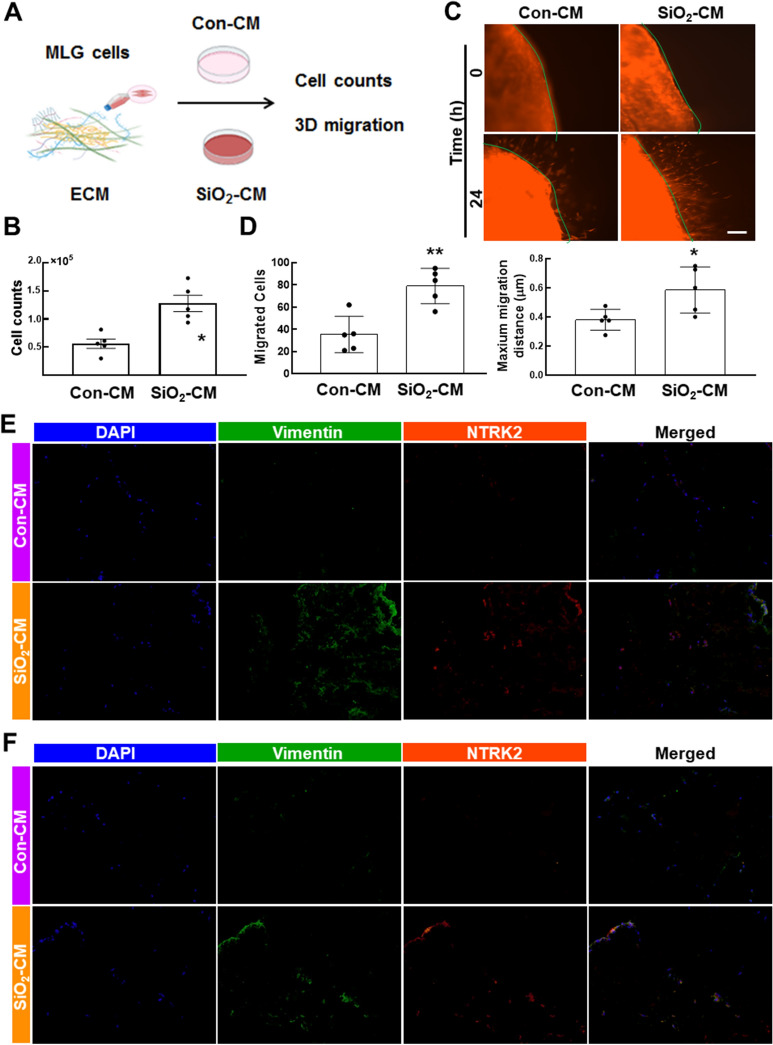
SiO_2_-CM promotes fibrosis and anoikis resistance in HPF-a cells growing in ECM. **A** Schematic diagram of fibroblasts transplanted to ECM after decellularization. **B** Cell counting showed the effect of SiO_2_-CM on MLG cell proliferation cultured in ECM (n = 5). *P < 0.05 compared with the control group. **C** In the 3D migration experiment, the migration of fibroblasts increased after 24 h of exposure to SiO_2_-CM. Scale bar = 275 μm. **D** Statistical results of the number of migrating fibroblasts in the 3D migration experiment and the maximum migration distance after 24 h of exposure to SiO_2_-CM (n = 5). *P < 0.05 compared with the control group and **P < 0.01 compared with the control group. Immunofluorescence staining showing the expression of the fibroblast markers Vimentin (**E**) and α-SMA (**F**) and the anoikis protein NTRK2 to detect the effect of SiO_2_-CM on MLG cells. Scale bar = 200 μm

Moreover, this pattern was not observed in epithelial cells or endothelial cells (Additional file 1: Figure S2B–E), reinforcing the unique mechanism of fibroblasts in pulmonary fibrosis. Colocalization of apoptosis (*Cas3* and *Bim1*, Fig. 2I and Additional file 1: Figure S2F–H) and proliferation (*Cdk1* and *Ccnb2*, Fig. 2J and Additional file 1: Figure S2I–K) markers with *Vimentin and Acta2* was detected to validate the in vitro results, and the levels of proliferation markers were increased significantly in the SiO_2_ groups. Then, MLG cells were transplanted back into decellularized ECM and exposed to a growth environment of cell nests (Fig. 3A). After three days, the cells were recovered and counted. The results showed that SiO_2_-CM promoted cell proliferation, which imitated the in vivo environment (Fig. 3B). Moreover, cell migration was increased in decellularized ECM treated with SiO_2_-CM (Fig. 3C, D). After three days of recellularization, we prepared ECM slices containing cells to determine whether MLG cells displayed anoikis resistance and fibrosis. Then, immunofluorescence staining showed that SiO_2_-CM induced the expression of Vimentin, α-SMA and TrkB. Based on these results, SiO_2_-CM activated cells and promoted fibrosis (Fig. 3E, F).

### ***ZC3H4 was involved in anoikis resistance in fibroblasts in response to SiO***_***2***_***-CM***

**Fig. 4:**
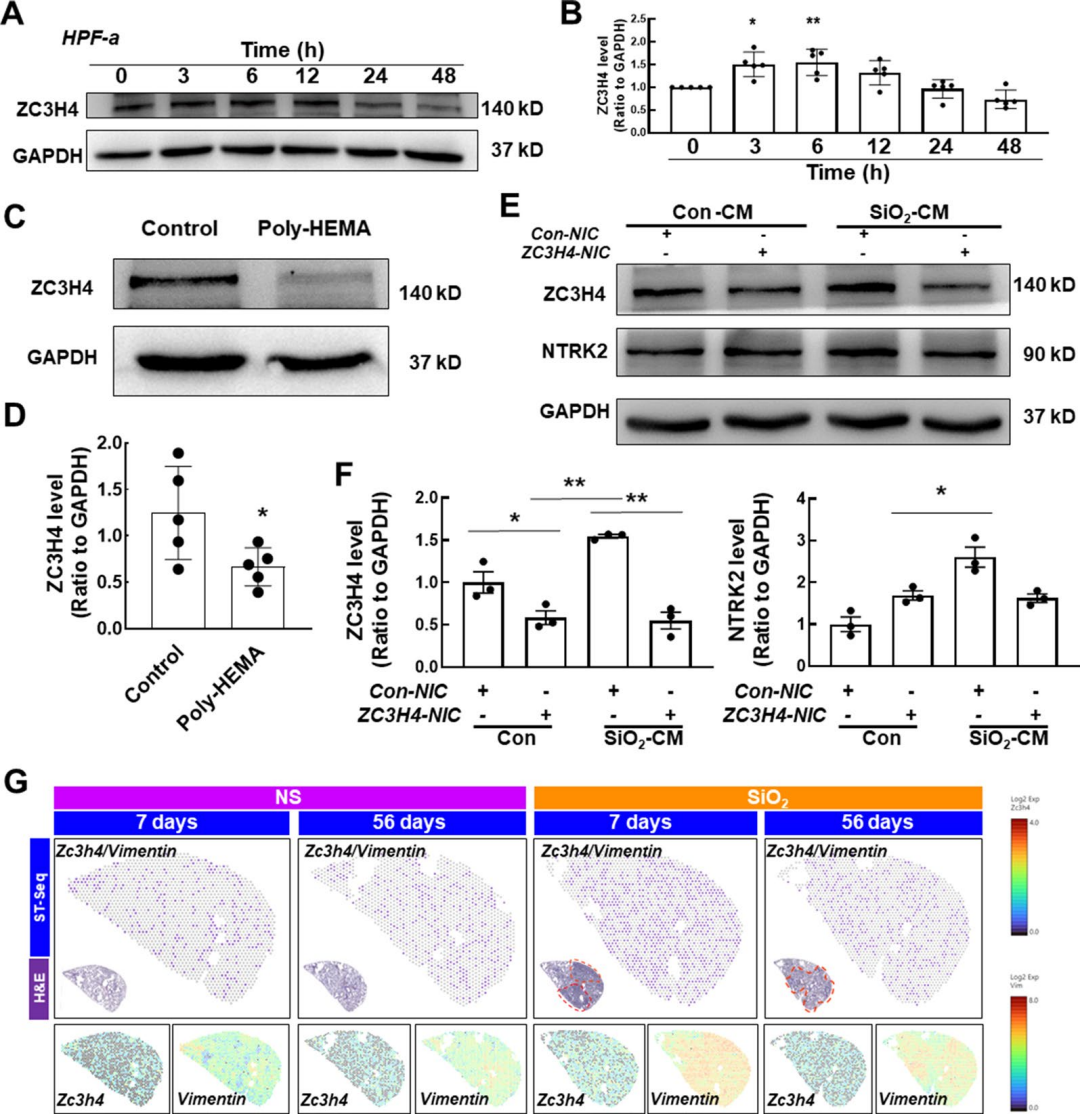
ZC3H4 is involved in anoikis resistance-mediated fibroblast activation in response to SiO_2_. **A** Representative western blot showing that macrophage CM upregulated ZC3H4 expression in HPF-a cells. **B** Densitometry analyses of five separate experiments suggested that macrophage CM upregulated ZC3H4 expression in HPF-a cells (n = 5); *P < 0.05 and **P < 0.001 compared with 0 h. **C** Representative western blot analysis suggesting that poly-HEMA attenuated CM-induced increases in ZC3H4 expression in HPF-a cells. **D** Densitometry analyses of five separate experiments (n = 5); *P < 0.05 compared with the control group. **E** Representative western blot showing the effects of ZC3H4-NIC transfection on ZC3H4 and NTRK2 expression in HPF-a cells. **F** Densitometry analyses of three separate experiments suggested that ZC3H4 NIC transfection affected CM-induced increases in ZC3H4 and TrkB expression in HPF-a cells (n = 3); *P < 0.05 and **P < 0.001 compared with the corresponding control group. **G** Spatial transcriptomics showing the coexpression of *Zc3h4* and *Vimentin*

We assessed the expression of ZC3H4, a member of the zinc finger protein family, in fibroblasts to obtain a better understanding of the molecular mechanism of anoikis resistance, since ZC3H4 was shown to be involved in pulmonary fibrosis [13, 16]. As shown in Fig. 4A and B, ZC3H4 expression was significantly increased and peaked at 6 h. We seeded fibroblasts in wells precoated with poly-HEMA to further determine the role of ZC3H4 in anoikis resistance, and ZC3H4 expression was significantly decreased compared to that in the control group (Fig. 4A, B). We next clarified the role of ZC3H4 in anoikis resistance using CRISPR/Cas9 technology to specifically knock down ZC3H4 in HPF-a cells (Additional file 1: Figure S3). As shown in Fig. 4E and F, the SiO_2_-CM-induced increase in NTRK2 levels in HPF-a cells was significantly reversed after the specific knockdown of ZC3H4, suggesting an important role for ZC3H4 in anoikis resistance. Moreover, the spatial transcriptomics results confirmed the increase in *Zc3h4* expression in fibroblasts, as indicated by its colocalization with *Vimentin* and *Acta2* (Fig. 4G and Additional file 1: Figure S3B).

### ***SiO***_***2***_***-CM induced endoplasmic reticulum stress but not autophagy in HPF-a cells***

**Fig. 5:**
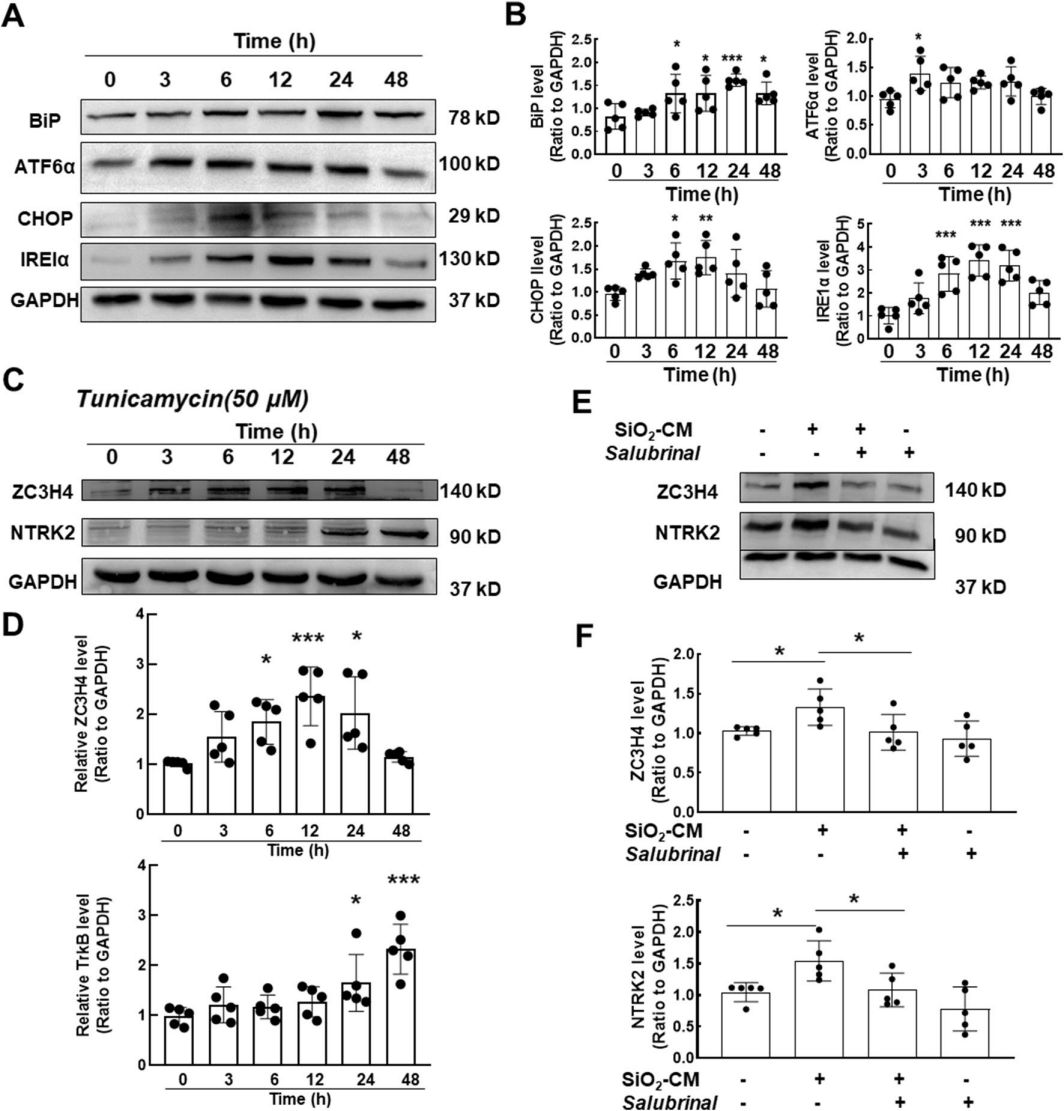
SiO_2_-induced pulmonary inflammation activates ERS, but not autophagy, in HPF-a cells. **A** Representative western blot showing that macrophage CM upregulated ERS-related protein expression in HPF-a cells. **B** Densitometry analyses of five separate experiments (n = 5); *P < 0.05, **P < 0.01, and ***P < 0.001 compared with 0 h. **C** HPF-a cells were treated with tunicamycin (an ERS agonist), and ZC3H4 and NTRK2 expression was upregulated in response to tunicamycin treatment. **D** Densitometry analyses of five separate experiments (n = 5); *P < 0.05 compared with the control. **E** HPF-a cells were treated with salubrinal (an ERS inhibitor) for 6 h, and western blot analysis showed that ZC3H4 and NTRK2 levels were downregulated in response to salubrinal treatment. **F** Densitometry analyses of five separate experiments (n = 5); *P < 0.05 compared with the control

Next, we investigated endoplasmic reticulum stress (ERS) and autophagy, since these processes play an important role in pulmonary fibrosis [15]. First, SiO_2_-CM induced significant increases in the expression of ERS markers (BiP, ATF6α, CHOP and IRE1α) (Fig. 5A, B). Moreover, the expression of the ZC3H4 protein first increased and then decreased, peaking at 6 h. The TrkB protein level continued to increase after stimulation with tunicamycin (Fig. 5C, D), which activates ERS. Based on the tunicamycin dose–response relationship experiment conducted by a previous research group [17], we set the concentration to 50 μM. Finally, when cells were pretreated with the ERS inhibitor salubrinal, the SiO_2_-CM-induced increases in ZC3H4 and NTRK2 levels were attenuated, suggesting that ERS participates in ZC3H4-mediated anoikis resistance in fibroblasts (Fig. 5E, F). Then, the expression of autophagy-related proteins (ATG5, BECN1 and LC3B) was also detected, and none of those markers showed significant changes (Additional file 1: Figure S4), excluding the participation of autophagy in anoikis resistance in fibroblasts.

### ***PI3K/MAPKs are involved in ZC3H4-mediated anoikis resistance in response to SiO***_***2***_

**Fig. 6:**
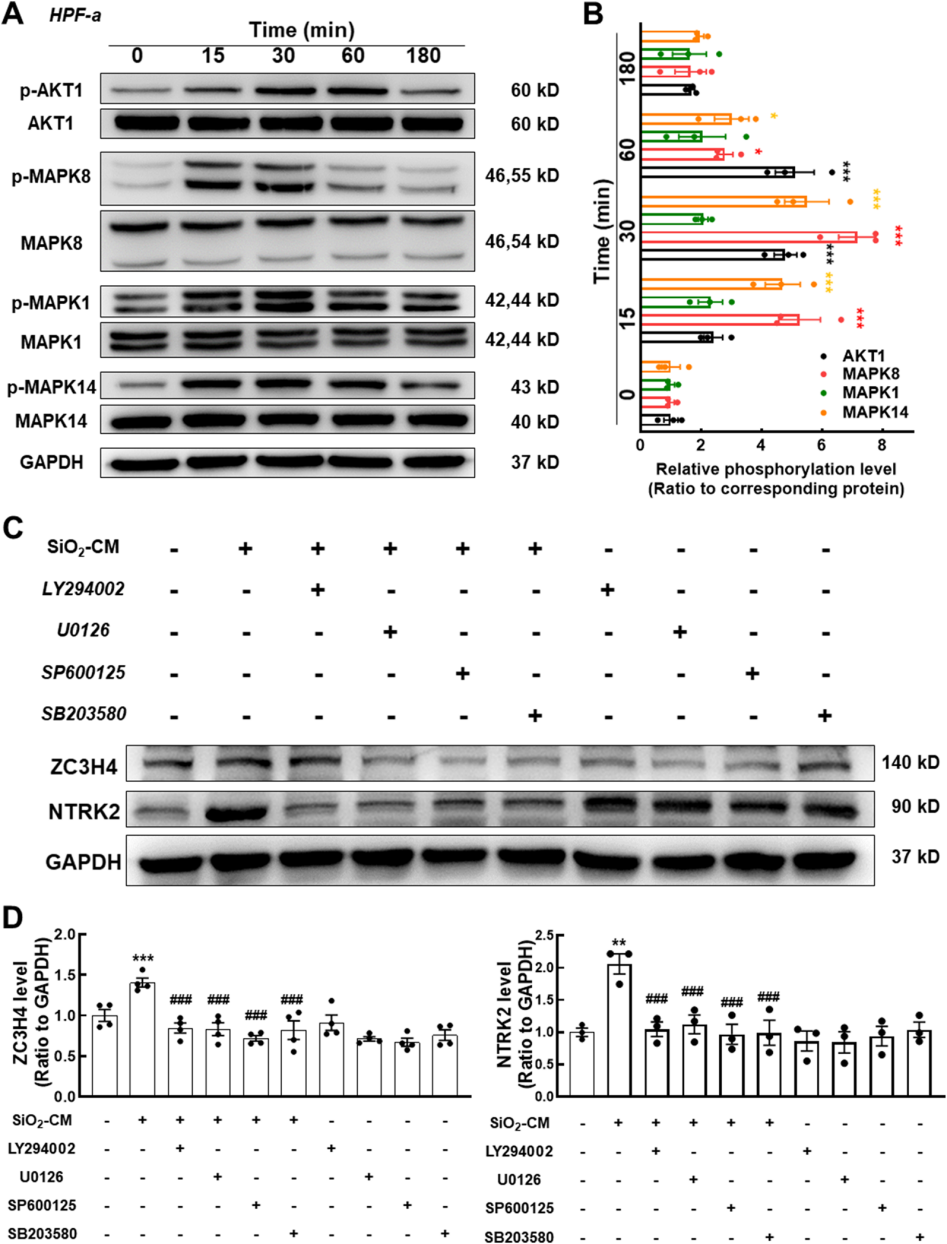
PI3K/MAPK signaling is involved in ZC3H4-mediated anoikis resistance in response to SiO_2_. **A** Representative western blot showing that macrophage CM increased the levels of PI3K/MAPK-related proteins in HPF-a cells. **B** Densitometry analyses of three separate experiments indicated that macrophage CM increased the levels of phosphorylated Akt, JNK, Erk, and P38 in HPF-a cells (n = 3); *P < 0.05 and ***P < 0.001 compared with the corresponding protein level detected at 0 h. **C** Representative western blot showing that CM-induced ZC3H4 and NTRK2 expression was attenuated by the pretreatment of HPF-a cells with a MAPK or PI3K/Akt inhibitor. **D** Densitometry analyses of ZC3H4 expression from 4 separate experiments and TrkB expression from 3 separate experiments. **P < 0.01 and ***P < 0.001 compared with the corresponding control group; ###P < 0.001 compared with the corresponding CM group

We also investigated prosurvival signals to further explore the molecular mechanism underlying the increase in ZC3H4 expression since these pathways were shown to have an important role in anoikis [18–20] and pulmonary fibrosis [15, 19, 21, 22]. The effects of SiO_2_-CM on the MAPK and PI3K/Akt pathways in fibroblasts were investigated. As shown in Fig. 6A and B, SiO_2_-CM induced a rapid and transient phosphorylation of MAPK8/JNK, MAPK1/ERK, MAPK14/p38 and AKT1 (0.25 h), which peaked at 30 or 60 min (Fig. 6A, B). We pretreated fibroblasts with specific inhibitors targeting JNK (SP600125), p38 (SB203580), MAPK14 (U0126) or PI3K (LY294002) to confirm the roles of the MAPK and PI3K/Akt pathways, and all of these inhibitors reversed the increases in ZC3H4 and NTRK2 levels induced by SiO_2_-CM (Fig. 6C, D). However, when LY294002 was used, ZC3H4 levels decreased to a lower degree, perhaps suggesting that ZC3H4 was mainly regulated by the MAPK signaling pathway. Thus, the PI3K and MAPK signaling pathways are involved in anoikis resistance by regulating ZC3H4 expression.

### The ECM of mice with silicosis was involved in the detachment of lung fibroblasts

**Fig. 7:**
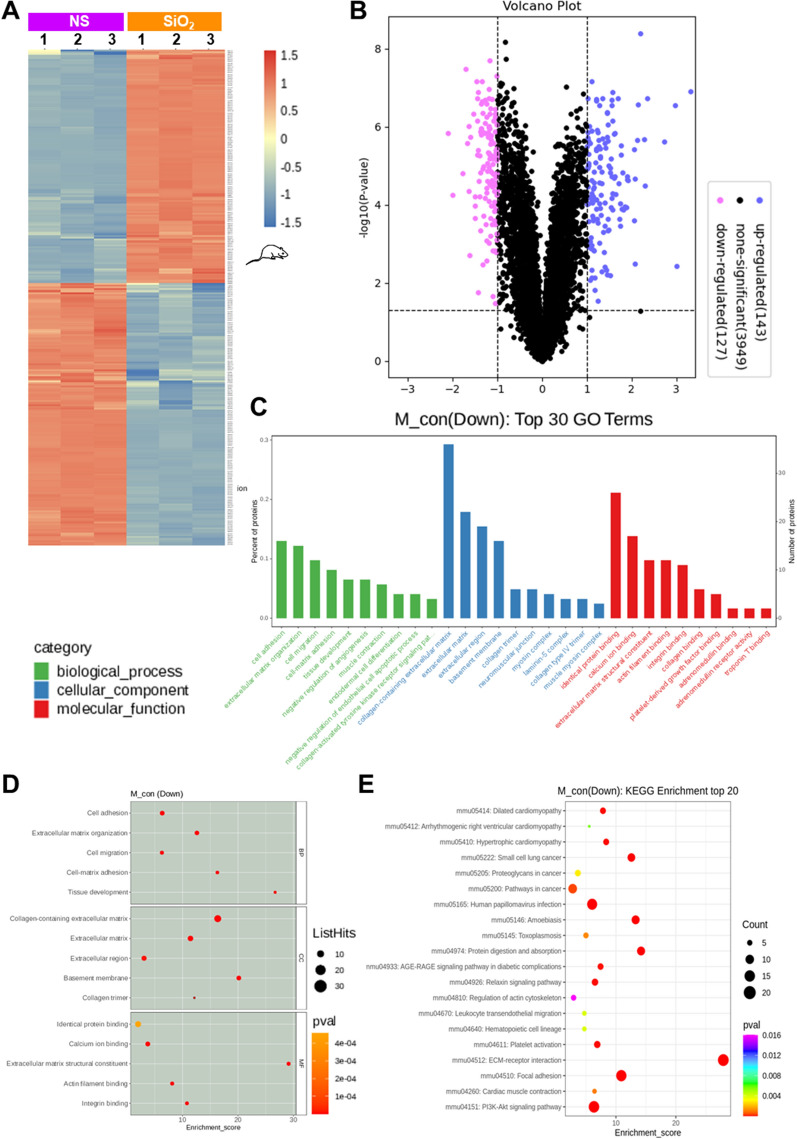
The ECM of mice with silicosis was involved in the detachment of lung fibroblasts. **A**, **B** According to the ECM proteomics results, 270 proteins showed differential expression (143 proteins were upregulated; 127 proteins were downregulated) in ECM derived from lung tissues of mice with silicosis (n = 3) with FC = 2. **C**, **D** GO analysis of the identified downregulated proteins: the top 30 GO entries were mainly involved in the biological process of cell adhesion, the major cellular components were related to collagen, and the main molecular function was the recognition of protein binding. **E** KEGG analysis of the downregulated proteins: among the top 20 enriched signaling pathways, mediating ECM receptor recognition and adhesion-related signaling were the most enriched pathways

**Fig. 8:**
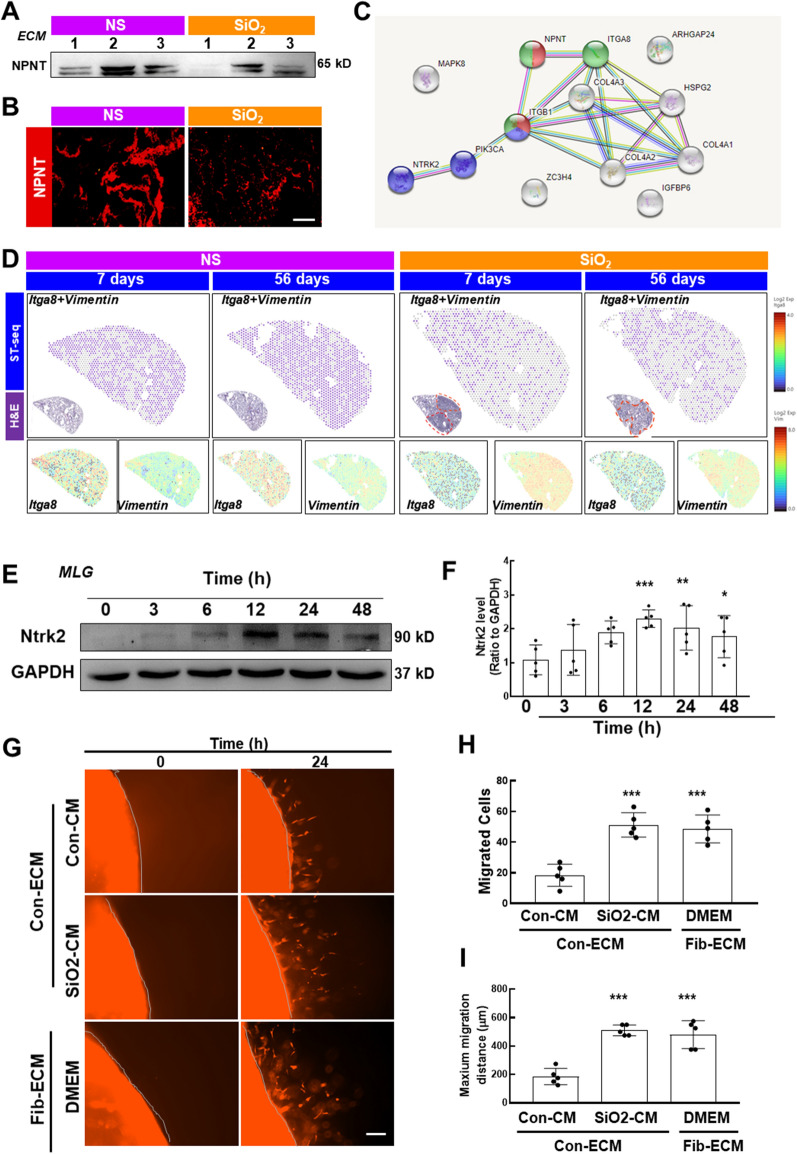
Detachment and apoptotic resistance of lung fibroblasts occurred independent of SiO2-induced pulmonary inflammation. **A** Representative western blot results showing the downregulation of NPNT expression in ECM protein samples. **B** Immunofluorescence staining was used to detect the differential expression of NPNT in the acellular ECM. Scale bar = 20 μm. **C** Protein–protein interaction network of the signaling pathways downstream of NPNT. **D** Spatial transcriptomics showing the coexpression of *Itga8* and *Vimentin*. **E** Representative western blot analysis suggesting that CM upregulated NTRK2 expression in MLG cells. **F** Densitometry analyses of five separate experiments suggested that SiO_2_-CM upregulated NTRK2 expression in MLG cells (n = 5); *P < 0.05 and **P < 0.01 compared with ZC3H4 expression at 0 h. **G** In the 3D migration experiment, the migration of fibroblasts was enhanced after 24 h of culture both in ECM (from NS-treated mice) exposed to SiO_2_-CM and in ECM from mice treated with SiO_2_ for 56 days. Scale bar = 275 μm. **H**, **I** Statistical results of the number of migrating fibroblasts in the 3D migration experiment and the maximum migration distance after 24 h of exposure to SiO_2_-CM (n = 5). ***P < 0.05 compared with the control group

Having determined the role of the apoptotic process in anoikis resistance, we then investigated the interaction in response to inappropriate ECM. The ECM in the lung not only provides structural support for cells but also plays important roles in organogenesis, injury repair, and homeostasis. Since anoikis is mainly caused by decreased adhesion between fibroblasts and the fibrotic ECM, we performed proteomic sequencing of the ECM isolated from the lungs of the saline- and SiO_2_-treated mice, and 147 differentially expressed proteins were upregulated and 123 differentially expressed proteins were downregulated (Fig. 7A, B). GO analysis showed that the downregulated proteins were mainly involved in the biological process of cell adhesion, and their main cellular components were collagen-related components (Fig. 7C, D). According to the KEGG signaling pathway enrichment analysis, the proteins with downregulated expression were highly enriched in mediating ECM-receptor recognition and adhesion-related signaling pathways (Fig. 7E). Based on these results, the ECM is involved in the loss of nest cells in the lung tissues of mice with silicosis. We analyzed the top 20 genes corresponding to the downregulated proteins to clarify the relationship between ECM and nested cells in lung tissues of mice with silicosis and found that most of the genes were related to adhesion, among which NPNT (a member of the epidermal growth factor (EGF)-like superfamily) showed the greatest downregulation. NPNT regulates integrin-mediated signaling through the interaction of its RGD motif with integrin α8β1 [23] and is also involved in regulating cell adhesion, differentiation, diffusion, and survival [24–26]. NPNT expression in the ECM was measured using western blotting (Fig. 8A) and immunofluorescence staining (Fig. 8B) to verify the proteomic results. Furthermore, downstream genes of NPNT were identified, among which the levels of *Itga8* (Fig. 8D and Additional file 1: Figure S5A), but not *Itgb1* (Additional file 1: Figure S5B), were decreased in the spatial transcriptomics analysis, indicating that this cascade is mediated by NPNT.

### ***SiO***_***2***_***-induced pulmonary inflammation facilitated anoikis resistance of fibroblasts attached to ECM***

Since the ECM proteomic results suggested that cell detachment might initiate the process of anoikis resistance, we must also clarify whether CM affects this process. For ethical reasons, the ECM was isolated from mice, and thus mouse pulmonary fibroblasts (MLGs) were used in subsequent experiments. The anoikis resistance pattern was confirmed by the detection of NTRK2 in MLG exposed to SiO_2_-CM from RAW264.7 cells treated with SiO_2_. As shown in Fig. 8E and F, SiO_2_-CM induced a similar increase in NTRK2 in MLG, which peaked at 12 h. Moreover, cell migration was increased both in normal decellularized ECM treated with SiO_2_-CM and in decellularized ECM isolated from mice exposed to SiO_2_ for 56 days (Fib-ECM) (Fig. 8G–I), which facilitated continuous fibrosis.

## Discussion

Pulmonary fibrosis is characterized by excessive fibroblast proliferation and abnormal collagen deposition [1, 4, 8, 19, 27–31] and has a pathological process similar to that of cancer. Although anoikis was shown to have an important role in cancer progression [10, 18, 32–34], the role of anoikis in pulmonary fibrosis remains unclear. The current study revealed a unique mechanism of anoikis in pulmonary fibroblasts, which depends on both resistance to anoikis and decreased adherence to the ECM, suggesting a tight connection between fibroblast proliferation and abnormal collagen deposition. These results indicate a vital role for anoikis resistance in fibroblast proliferation during pulmonary fibrosis.

In the current study, silica was applied to establish the pulmonary fibrosis model, which is a typical form of chronic pulmonary fibrosis [35, 36] that mimics the clinical symptoms of fibrosis, such as silicosis. Silicosis is one of the major occupational diseases worldwide and is caused by long-term inhalation of SiO_2_, with pulmonary fibrosis as the outcome [37]. Although many studies have been performed, the mechanism of pulmonary fibrosis in silicosis has not yet been established. China has the largest number of patients with silicosis in the world, and due to the large population, the number of these patients is increasing annually. Therefore, the elucidation of the etiology of silicosis is still underway. Silica-induced pulmonary fibrosis is related to the particle size of inhaled SiO_2_ [38]. Compared with most nontoxic nano-SiO_2_ particles, micrometer-sized SiO_2_ particles have a strong ability to induce fibrosis, with fibrotic nodules appearing after 30–60 days in exposed mice. Unfortunately, micrometer-sized SiO_2_ particles are more common pollutants in occupational environments [35]. In this study, SiO_2_ particles with a diameter of approximately 5 μm were used as the main irritant, which helped clarify the specific process of pulmonary fibrosis with a similar mechanism.

Fibroblast proliferation is the main pathological process in pulmonary fibrosis [1, 39, 40] and may be due to either an increase in cell number or a decrease in cell death. A recent study suggested a direct effect of SiO_2_ on fibroblast proliferation, which was mainly caused by a decrease in the apoptosis of fibroblasts via ERS [41]. Interestingly, CM from macrophages exposed to SiO_2_ did not exert any significant effect on fibroblast apoptosis, indicating a different effect of the inflammatory environment compared to the direct effect of SiO_2_ on fibroblasts. Apoptosis mainly occurs in pulmonary macrophages after phagocytosis of silica particles (also known as dust cells), followed by an inflammatory cascade at the early stage. Dust cells undergo apoptosis and release SiO_2_ particles again, forming a vicious cycle with the release of inflammatory factors, which leads to alveolar epithelial injury and fibroblast activation [30, 42]. Furthermore, SiO_2_ particles in the alveoli that directly contact epithelial cells cause edema and necrosis [29]. Loss of epithelial cells leads to exposure of the lung interstitium, upon which lung fibroblasts migrate outward and are activated into myofibroblasts through the direct and indirect activities of SiO_2_ [40]. Obviously, multiple mechanisms, such as classical apoptosis, necrosis, or ferroptosis, are involved in the excessive proliferation of fibroblasts. Notably, as a type of mesenchymal cell, fibroblasts are strongly affected by the environment, such as the ECM. As shown in the current study, inflammatory factors released by macrophages exacerbated apoptosis in detached fibroblasts, indicating that excess proliferation of fibroblasts should be a comprehensive result of direct and indirect effects produced by either macrophages or ECM. Moreover, the specific increase in the level of the anoikis resistance marker NTRK2 suggested a unique interaction between fibroblasts and ECM during pulmonary fibrosis.

Based on accumulating evidence, the ECM regulates cell function, fate and phenotype under physiological conditions, while the composition and function of the ECM are obviously disordered in pathological tissue remodeling [43]. Anoikis is a physiological protective mechanism to prevent excessive proliferation of fibroblasts that have detached from the ECM [5, 34], which may be caused by disruption of the integrin signaling pathway in fibroblasts [32] or changes in the components within the fibrotic ECM. Few reports are available on the global changes in proteins in the ECM during pulmonary fibrosis, and thus proteomics was utilized to analyze the change in the protein profile of the ECM after SiO_2_ exposure. As expected, various proteins involved in mediating cell-ECM adhesions were downregulated, indicating the initiation of anoikis and suggesting that detachment and proliferation in anoikis resistance experienced by fibroblasts are two independent events that must be investigated separately.

ZC3H4, a novel member of the zinc finger protein family, has been shown to play a key role in anoikis resistance in fibroblasts, since a recent study suggested a role for zinc finger proteins in anoikis under different conditions [33, 34]; for example, ZNF32 and ZNF304 promoted abnormal tissue repair and mitigated tumor cell metastasis via anoikis resistance. Accordingly, both ZC3H12A and ZC3H4 are involved in the progression of pulmonary fibrosis [12, 13, 15, 16, 21], but the connection to anoikis is still unclear. In this study, ZC3H4 was observed to regulate the anoikis resistance of fibroblasts and participate in pulmonary fibrosis. Since both autophagy [44–46] and ERS [47–49] play important roles in tissue fibrosis, as well as the regulation of abnormal protein expression, the relationship between ZC3H4 and autophagy/ERS was investigated. In addition, the ZC3H4 signaling pathway was verified, in which MAPK/PI3K signaling promoted anoikis resistance, followed by pulmonary fibrosis.

**Fig. 9:**
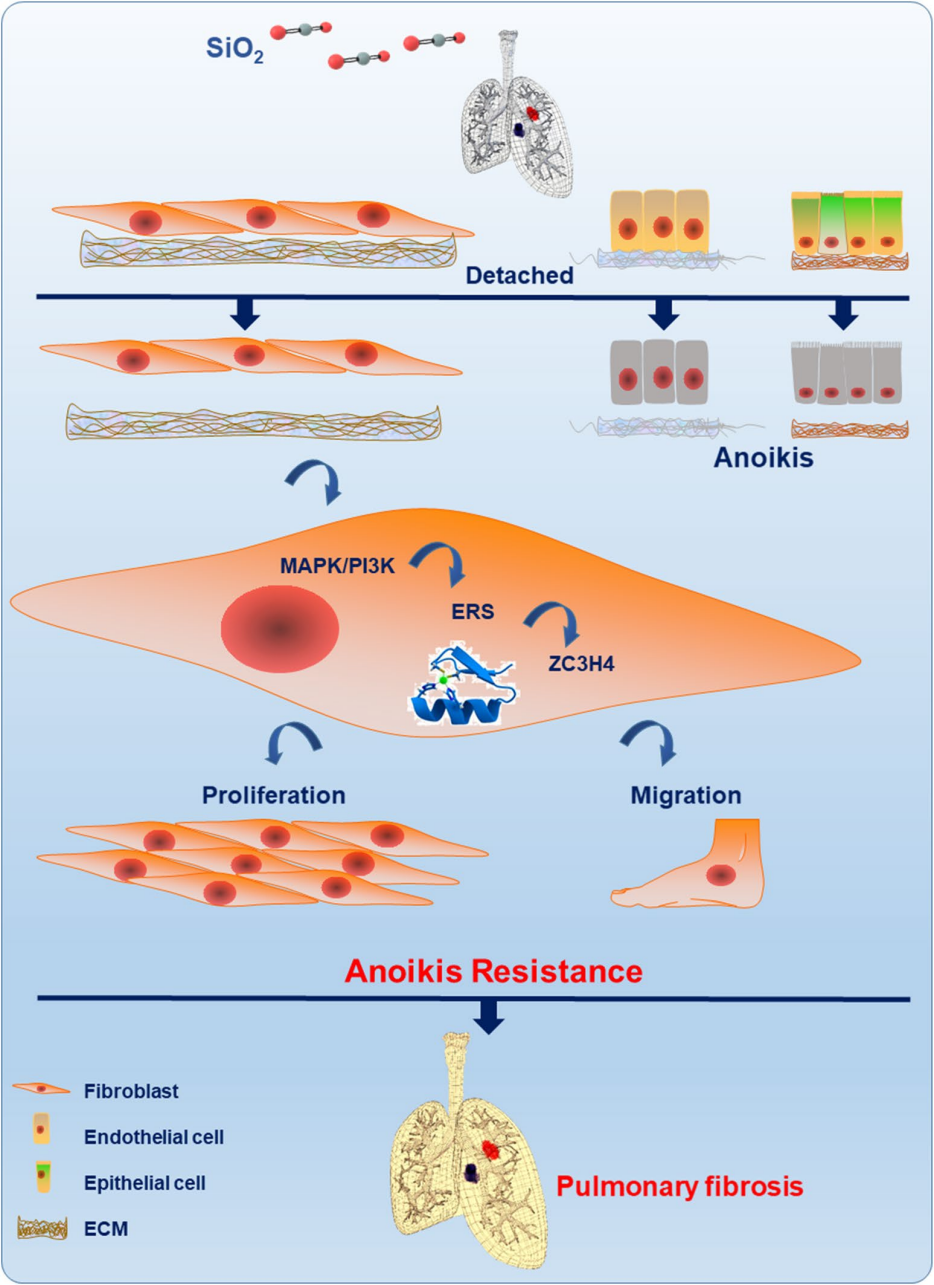
The mechanism of anoikis resistance induced by SiO_2_-mediated pulmonary inflammation in fibroblasts. In fibroblasts exposed to SiO_2_, adhesion to the ECM was weakened, and detachment occurred. In addition, the prosurvival PI3K/MAPK signaling pathway was activated, which subsequently led to increased ZC3H4 expression, enhancing anoikis resistance in fibroblasts. Apoptotic resistance of fibroblasts and activation of differentiated myofibroblasts resulted in increased proliferation, migration, and collagen synthesis

In summary, SiO_2_ induced the synergistic activation of macrophage-derived inflammatory factors that promoted fibroblasts that had detached from the ECM to undergo proliferation in a process named anoikis resistance, followed by persistent and irreversible pulmonary fibrosis. ZC3H4 mediated anoikis resistance by activating ERS and the MAPK/PI3K signaling pathway (Fig. 9). The results of this study suggest that anoikis resistance is strongly associated with fibrosis and exhibits abnormal activity during the repair of abnormal lung tissue.

Our study showed that fibroblasts exhibit detachment and anoikis resistance in lung tissues during fibrosis. The zinc finger protein ZC3H4 regulates the development of anoikis resistance in fibroblasts, and its expression is increased during fibrosis. The PI3K and MAPK signaling pathways are activated during pulmonary fibrosis, and anoikis resistance is regulated by ZC3H4 (Fig. 9). Thus, a combination therapy targeting key inflammatory factors, growth-promoting factors, and epigenetic modifications may be the most successful strategy for treating highly complex and devastating fibrotic diseases.

## Supplementary Information

**Additional file 1: Figure S1**. (A) Statistical analysis of Masson’s trichrome staining of the fibrotic area. (n=5); *p < 0.001 compared with the NS group. (B) Cell viability was detected using the CCK-8 assay after the three types of cells were treated with Con-CM and SiO2-CM for 6 h. Statistical analysis of cell viability. (n=5); *p < 0.005 compared with the Con-CM group. (C) The expression levels of TrkB and Bcl2l11 were detected using Western blotting after HPF-a cells were treated with Con-CM and SiO2-CM for 24 h. Statistical analysis of cell viability (n=5); *p < 0.05 compared with the Con-CM group. (D) The expression levels of TrkB and Bcl2l11 were detected using western blotting after BEAS-2B cells were treated with Con-CM and SiO2-CM for 24 h. Statistical analysis of cell viability (n=5); *p < 0.05 compared with the Con-CM group. (E) The expression levels of TrkB and Bcl2l11 were detected using western blotting after HPMECs were treated with Con-CM and SiO2-CM for 24 h. Statistical analysis of cell viability (n=5); *p < 0.05 compared with the Con-CM group. (F). Immunofluorescence staining showing the expression of the mesenchymal markers α-SMA and Col1a1 and the anoikis marker TrkB. Scale bar=200 μm. (H). Immunofluorescence staining showing the expression of the epithelial marker Cdh1 and the anoikis marker TrkB. Scale bar=100 μm. (I). Immunofluorescence staining showing the expression of the endothelial cell marker Cdh5 and the anoikis marker TrkB. Scale bar=100 μm. (J) Spatial transcriptomics showing the coexpression of *Ntrk2* and *Acta2*. **Figure S2**. (A) Morphology of HPF-a cells after suspension culture for 48 h. Scale bar=100 μm. (B) BEAS-2B cells were suspended for 48 hours, cocultured with CM for 24 h, and double stained with Annexin V and PI. The sum of the counts in Q2-1, Q2-2 and Q2-4 was defined as the number of apoptotic cells. (C) HPMECs were treated as described in (B). The sum of the counts in Q2-1, Q2-2 and Q2-4 was defined as the number of apoptotic cells. (D) Statistical analysis of three independent experiments of BEAS-2B cell apoptosis assessed using flow cytometry (n=3); *p < 0.05 compared with the attached group. (E). Statistical analysis of three independent experiments of HPMEC apoptosis assessed using flow cytometry (n=3); *p < 0.05 compared the attached group. (F) Spatial transcriptomics showed the coexpression of *Bim* and *Acta2*. (G) Spatial transcriptomics showed the coexpression of *Cas3* and *Acta2*. (H) Spatial transcriptomics showed the coexpression of *Cas3* and *Vimentin*. (I) Spatial transcriptomics showed the coexpression of *Cdk1* and *Acta2*. (J) Spatial transcriptomics showed the coexpression of *Ccnb2* and *Acta2*. (K) Spatial transcriptomics showed the coexpression of *Ccnb2* and *Vimentin*. **Figure S3**. (A) Representative western blot showing the transfection efficiency of ZC3H4-NICs in HPF-a cells. (B) Spatial transcriptomics showed the coexpression of *Zc3h4* and *Acta2*. **Figure S4**. The expression of autophagy-related proteins (ATG5, BECN1 and LC3B) after CM stimulation of HPF-a cells. **Figure S5**. (A) Spatial transcriptomics showed the coexpression of *Itga8* and *Acta2*. (B) Spatial transcriptomics showed the coexpression of *Itgb1* and *Vimentin*.

## Data Availability

All of the relevant raw data and materials are freely available to any investigator upon request.

## Abbreviations

FCM: Flow cytometry
SiO_2_: Silicon dioxide
ECM: Extracellular matrix
MCPIP1: Monocyte chemotactic protein-1-induced protein 1
CM: Conditioned medium
TrkB: NTRK2
BEAS-2B: Human bronchial epithelial cell line
HPMECs: Human pulmonary microvascular endothelial cells
HPF-a: Human pulmonary fibroblasts-adult
ERS: Endoplasmic reticulum stress
MLG: Mouse pulmonary fibroblasts
MCP-1: Monocyte chemoattractant protein-1
CCL2: C–C chemokine ligand 2
CCR2: C–C chemokine receptor 2
DMEM: Dulbecco's modified Eagle's medium
NS: Normal saline
FBS: Fetal bovine serum
NGS: Normal goat serum
BALF: Bronchoalveolar lavage fluid
PCA: Principal component analysis
poly-HEMA: Poly-2-hydroxyethyl methacrylate
