# Supplementary material for: A missing piece of the puzzle in pulmonary fibrosis: anoikis resistance promotes fibroblast activation
Source: Cell Biosci. 2022 Feb 25;12:21. doi: 10.1186/s13578-022-00761-2 (PMC8881884; doi:10.1186/s13578-022-00761-2)
Supplement: Supplementary file 1 — Additional file 1: Figure S1. (A) Statistical analysis of Masson’s trichrome staining of the fibrotic area. (n=5); *p < 0.001 compared with the NS group. (B) Cell viability was detected using the CCK-8 assay after the three types of cells were treated with Con-CM and SiO2-CM for 6 h. Statistical analysis of cell viability. (n=5); *p < 0.005 compared with the Con-CM group. (C) The expression levels of TrkB and Bcl2l11 were detected using Western blotting after HPF-a cells were treated with Con-CM and SiO2-CM for 24 h. Statistical analysis of cell viability (n=5); *p < 0.05 compared with the Con-CM group. (D) The expression levels of TrkB and Bcl2l11 were detected using western blotting after BEAS-2B cells were treated with Con-CM and SiO2-CM for 24 h. Statistical analysis of cell viability (n=5); *p < 0.05 compared with the Con-CM group. (E) The expression levels of TrkB and Bcl2l11 were detected using western blotting after HPMECs were treated with Con-CM and SiO2-CM for 24 h. Statistical analysis of cell viability (n=5); *p < 0.05 compared with the Con-CM group. (F). Immunofluorescence staining showing the expression of the mesenchymal markers α-SMA and Col1a1 and the anoikis marker TrkB. Scale bar=200 μm. (H). Immunofluorescence staining showing the expression of the epithelial marker Cdh1 and the anoikis marker TrkB. Scale bar=100 μm. (I). Immunofluorescence staining showing the expression of the endothelial cell marker Cdh5 and the anoikis marker TrkB. Scale bar=100 μm. (J) Spatial transcriptomics showing the coexpression of Ntrk2 and Acta2. Figure S2. (A) Morphology of HPF-a cells after suspension culture for 48 h. Scale bar=100 μm. (B) BEAS-2B cells were suspended for 48 hours, cocultured with CM for 24 h, and double stained with Annexin V and PI. The sum of the counts in Q2-1, Q2-2 and Q2-4 was defined as the number of apoptotic cells. (C) HPMECs were treated as described in (B). The sum of the counts in Q2-1, Q2-2 and Q2-4 was defined as the number of apopto [file 13578_2022_761_MOESM1_ESM.docx]

**A missing piece of the puzzle in pulmonary fibrosis: anoikis resistance promotes fibroblast activation**

**Running title:** Anoikis resistance promotes fibroblast activation

Juan Yin, Jing Wang, Xinxin Zhang, Yan Liao, Wei Luo, Sha Wang, Jiawei Ding, Jie Huang, Mengling Chen, Shencun Fang, and Jie Chao

**Table of contents**

Materials and methods 2-4

Supplementary Figure S1 5-7

Supplementary Figure S2 8-11

Supplementary Figure S3 12

Supplementary Figure S4 13

Supplementary Figure S5 14

**Materials and methods**

***1. Western blot analysis***

Western blot analysis was performed as described in a previous study [14]. Primary antibodies against ZC3H4 (1:1000), TrkB (1:800), Bim (1:1000), Caspase-3 (1:500), cleaved Caspase-3 (1:500), JNK (1:1000), p-JNK (1:1000), Akt (1:1000), p-Akt (1:1000), P38 (1:1000), p-P38 (1:1000), Bax (1:1000), Npnt (1:1000) and Bcl-xL (1:1000) were used. All western blots shown are representative of three or more independent experiments. The protein band intensities were quantiﬁed using ImageJ v1.48 software.

***2. Immunofluorescence staining***

The mouse lung tissue was embedded in OCT compound. When the OCT gel solidified, 5- to 10-μm-thick frozen tissue slices were obtained by sectioning and placed on slides. The edge of the slides was surrounded with hydrogel, and the sections were fixed with 4% paraformaldehyde. The OCT compound was removed by rinses with PBS before the sections were blocked with 10% normal goat serum in 0.3% Triton X-100 for 2 h at room temperature followed by an incubation with primary antibodies against ZC3H4, TrkB or Npnt at 4°C overnight. The next day, the tissue was incubated with the appropriate fluorescent dye-conjugated secondary antibodies (Alexa Fluor, Thermo Fisher Scientific). A fluorescence microscope (Olympus IX70, Olympus America, Inc., Center Valley, PA, USA) was used to observe the tissue sections.

***3. Cell viability***

Cells were seeded in 96-well plates at a density of 5000 per well and stimulated with macrophage conditioned medium (CM). After the reaction, CCK-8 reagent (APExBIO, USA) was added at a ratio of 10:1 and incubated with the cells for 30 min in the dark. Finally, the absorbance was measured at 450 nm with a microplate reader (BioTek, USA)

***4. Quantitative reverse transcription-polymerase chain reaction (qRT–PCR)***

Real-time quantitative PCR (qRT–PCR) was performed to determine the relative expression of circ-ZC3H4 and *Zc3h4* mRNA. Total RNA was extracted from HPF-a cells with TRIzol reagent (Invitrogen) according to the manufacturer’s instructions. After the extraction of total RNA, the concentration was measured using a NanoDrop One spectrophotometer (Thermo Fisher Scientific). The volumes of the samples were adjusted, and 300 ng or 400 ng of RNA were reverse-transcribed into cDNAs, which were used as templates for real-time qRT–PCR. The cycle threshold (Ct) and ΔCT values were analyzed. The ΔΔCT quantification method was applied using Opticon Monitor software (Bio-Rad). The relative quantitative expression of each mRNA was normalized to that of GAPDH (endogenous reference gene).

***5. Flow cytometry analysis of apoptotic cells***

FCM was performed according to the manufacturer’s instructions (Keygentec, KGA108, China). The stained cells were incubated in the dark for at least 15 min at 4°C, and the percentages of apoptotic cells were quantiﬁed using a FACSCalibur ﬂow cytometer within 1 h of staining.

***6. Nested matrix model and cell migration assay***

We used a three-dimensional (3D) migration model described in a previous study, with some modifications [16]. For the nested attachment matrix, standard fibroblasts were transplanted into ECM and incubated for 48 h in the attachment state with DMEM containing 5% FBS. The ECM was then removed from the culture well and placed into 60 μL of fresh acellular collagen matrix solution centered around the scratch area (12 mm diameter). The newly metastatic fibroblast-populated collagen matrix (FPCM) was then covered with 140 μL of acellular collagen matrix solution. The matrix system was polymerized at 37°C with 5% CO_2_ for 1 h. Then, 1 mL of DMEM containing 10% FBS was added to the well. In these experiments, we used CM derived from THP-1 macrophages. Fresh medium was mixed with the prepared CM at a 1:1 ratio. The migration of cells from the ECM to the acellular matrix was observed. Cell migration observed at 6, 12, and 24 h by fluorescence microscopy was compared with that observed at 0 h. Digital images of the interface between the ECM and the acellular matrix were captured using an EVOS FL cell imaging microscope (Thermo Fisher Scientific). The migration of lung fibroblasts (PFBs) from the ECM was quantified by counting the number of cells that significantly migrated from the nested matrix to the acellular matrix. The maximum migration distance was quantified by determining the cell that had traveled the maximum distance from the nested matrix to the element-free matrix and measuring that distance. The average number of cells in each field that migrated from the nested matrix and the maximum migration distance of cells in each field were recorded.

**Figure S1**


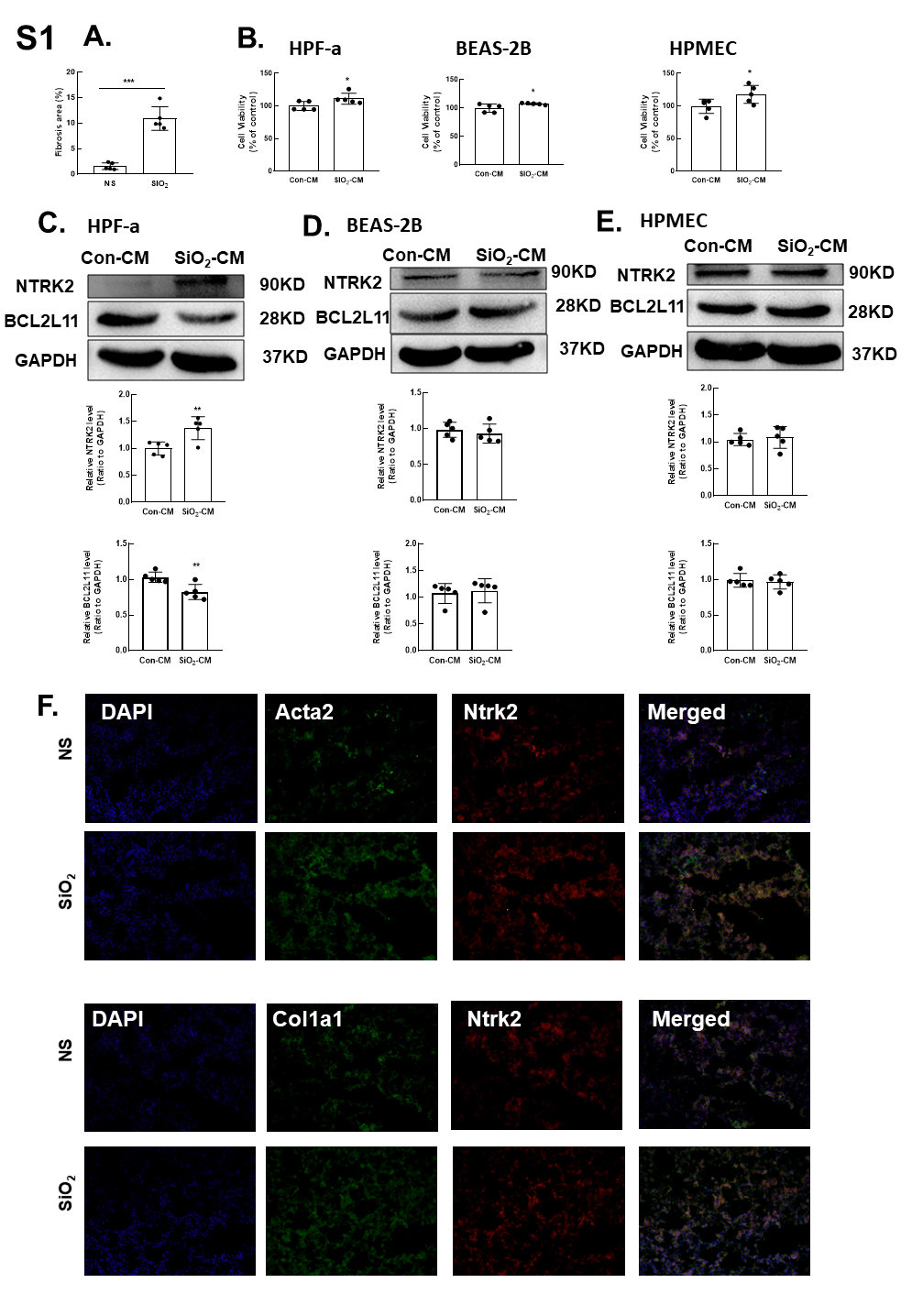

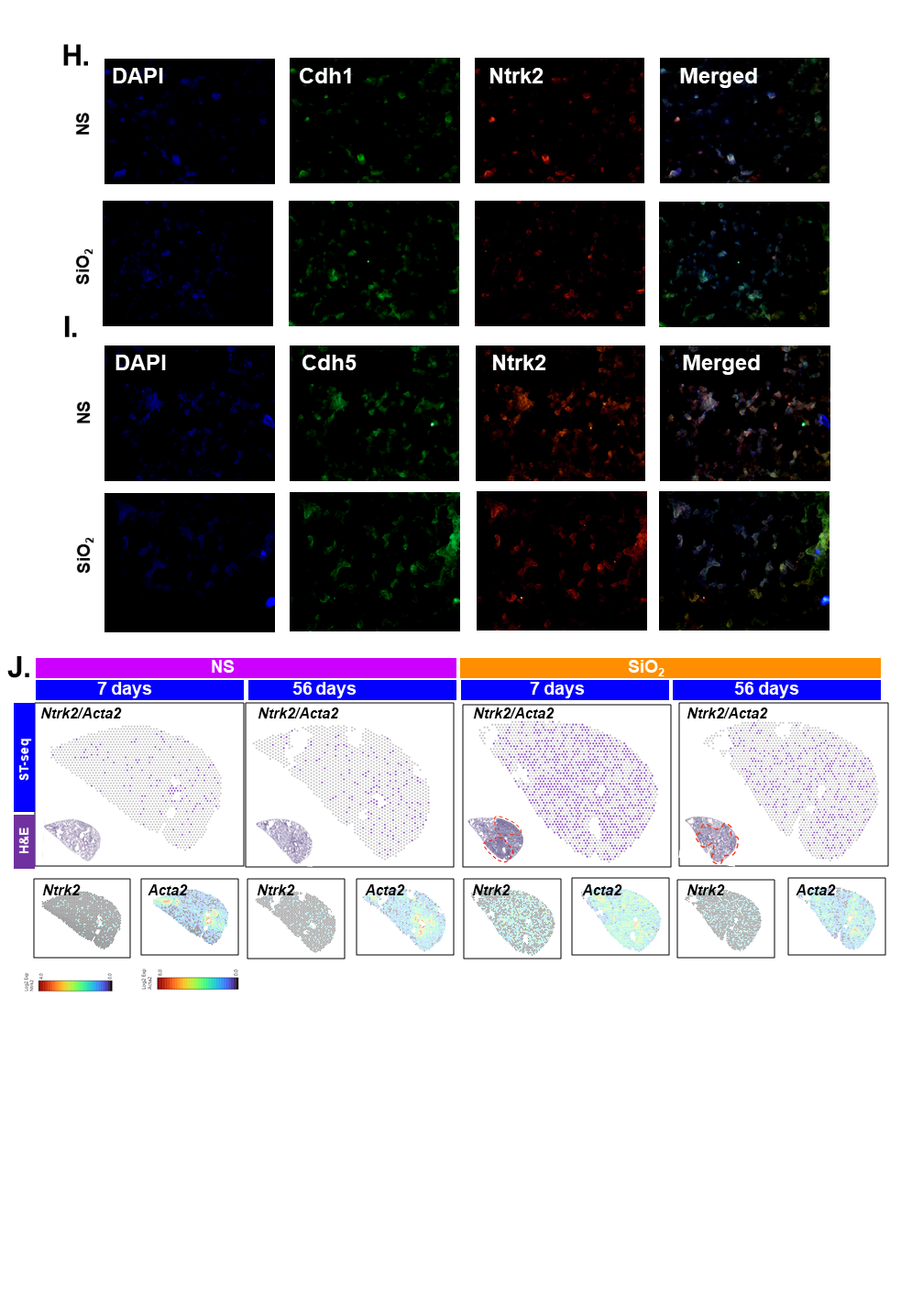


**Supplementary Figure S1.** (A) Statistical analysis of Masson’s trichrome staining of the fibrotic area. (n=5); *p < 0.001 compared with the NS group. (B) Cell viability was detected using the CCK-8 assay after the three types of cells were treated with Con-CM and SiO_2_-CM for 6 h. Statistical analysis of cell viability. (n=5); *p < 0.005 compared with the Con-CM group. (C) The expression levels of TrkB and Bcl2l11 were detected using Western blotting after HPF-a cells were treated with Con-CM and SiO_2_-CM for 24 h. Statistical analysis of cell viability (n=5); *p < 0.05 compared with the Con-CM group. (D) The expression levels of TrkB and Bcl2l11 were detected using western blotting after BEAS-2B cells were treated with Con-CM and SiO_2_-CM for 24 h. Statistical analysis of cell viability (n=5); *p < 0.05 compared with the Con-CM group. (E) The expression levels of TrkB and Bcl2l11 were detected using western blotting after HPMECs were treated with Con-CM and SiO_2_-CM for 24 h. Statistical analysis of cell viability (n=5); *p < 0.05 compared with the Con-CM group. (F). Immunofluorescence staining showing the expression of the mesenchymal markers α-SMA and Col1a1 and the anoikis marker TrkB. Scale bar=200 μm. (H). Immunofluorescence staining showing the expression of the epithelial marker Cdh1 and the anoikis marker TrkB. Scale bar=100 μm. (I). Immunofluorescence staining showing the expression of the endothelial cell marker Cdh5 and the anoikis marker TrkB. Scale bar=100 μm. (J) Spatial transcriptomics showing the coexpression of *Ntrk2* and *Acta2*.

**Figure S2**

**
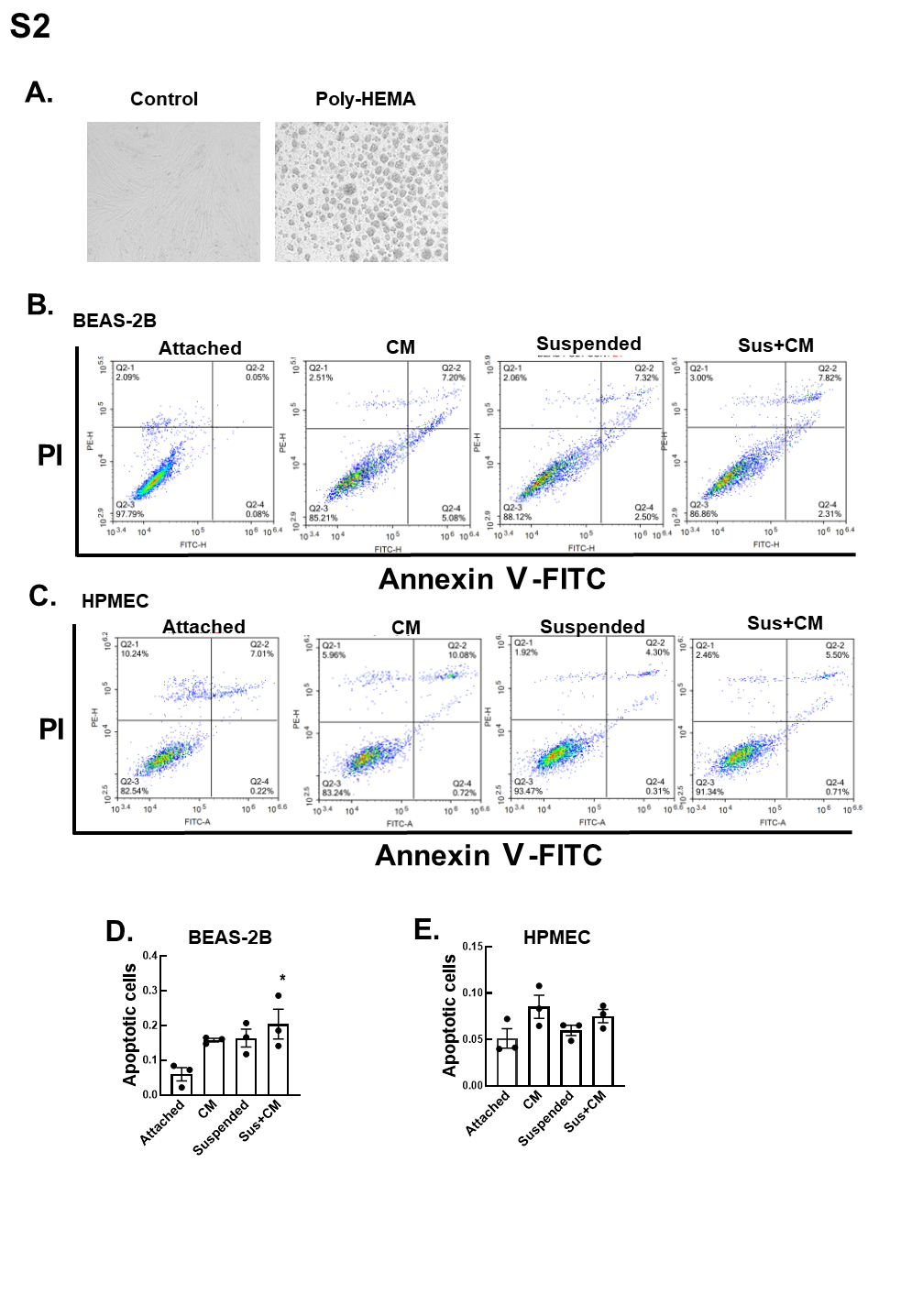

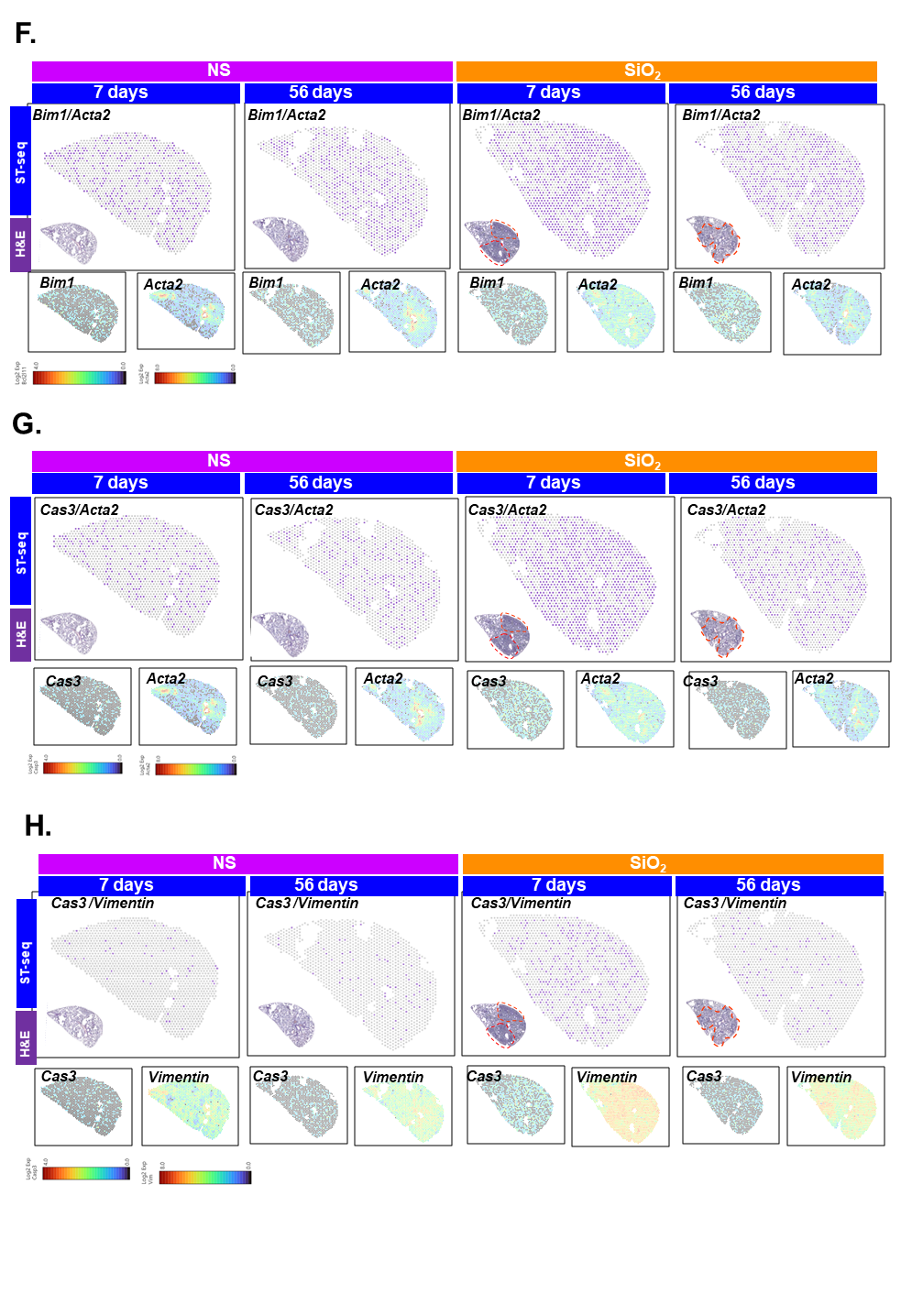

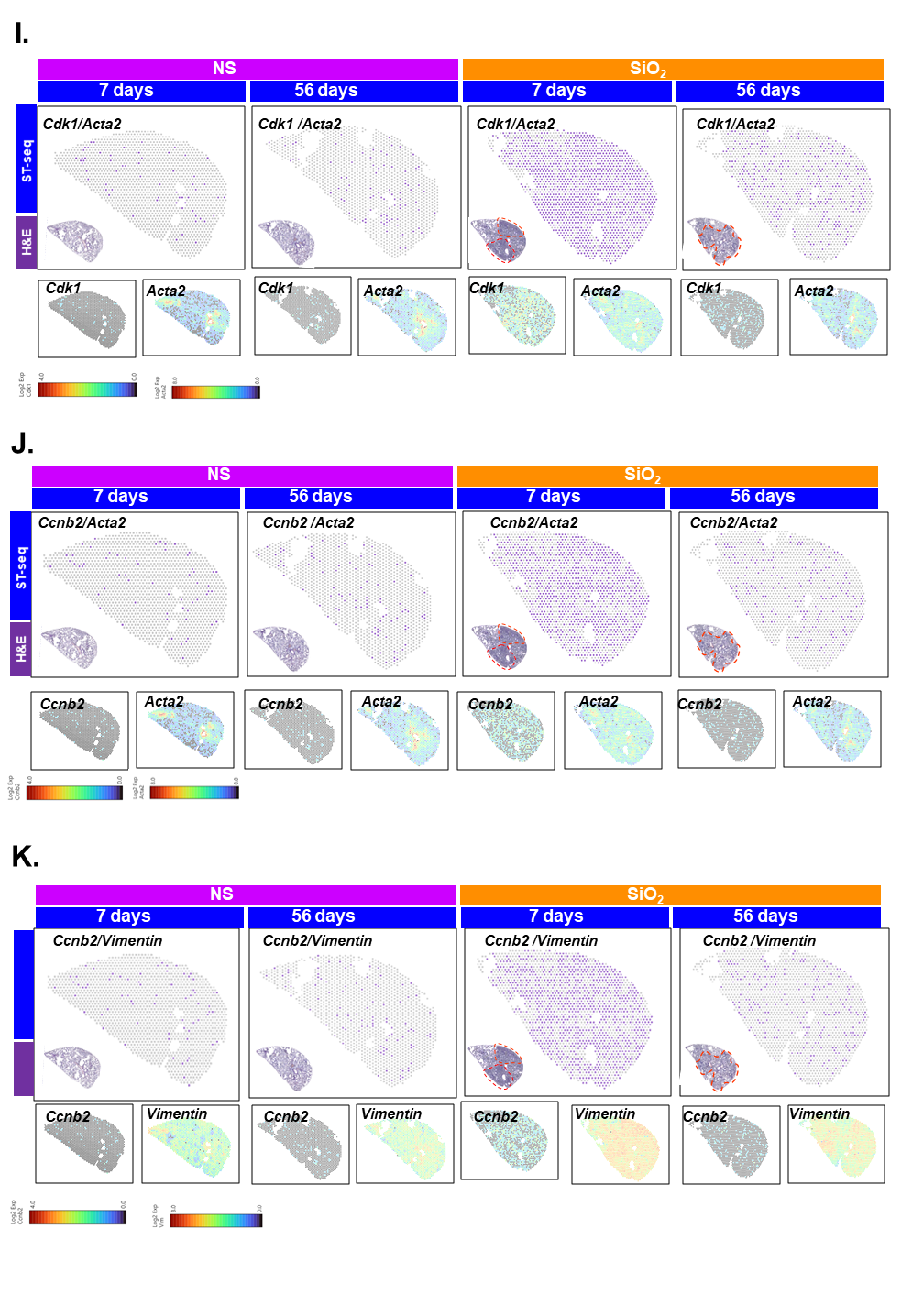
**

**Supplementary Figure S2.** (A) Morphology of HPF-a cells after suspension culture for 48 h. Scale bar=100 μm. (B) BEAS-2B cells were suspended for 48 hours, cocultured with CM for 24 h, and double stained with Annexin V and PI. The sum of the counts in Q2-1, Q2-2 and Q2-4 was defined as the number of apoptotic cells. (C) HPMECs were treated as described in (B). The sum of the counts in Q2-1, Q2-2 and Q2-4 was defined as the number of apoptotic cells. (D) Statistical analysis of three independent experiments of BEAS-2B cell apoptosis assessed using flow cytometry (n=3); *p < 0.05 compared with the attached group. (E). Statistical analysis of three independent experiments of HPMEC apoptosis assessed using flow cytometry (n=3); *p < 0.05 compared the attached group. (F) Spatial transcriptomics showed the coexpression of *Bim* and *Acta2*. (G) Spatial transcriptomics showed the coexpression of *Cas3* and *Acta2*. (H) Spatial transcriptomics showed the coexpression of *Cas3* and *Vimentin*. (I) Spatial transcriptomics showed the coexpression of *Cdk1* and *Acta2*. (J) Spatial transcriptomics showed the coexpression of *Ccnb2* and *Acta2*. (K) Spatial transcriptomics showed the coexpression of *Ccnb2* and *Vimentin*.

**Figure S3**


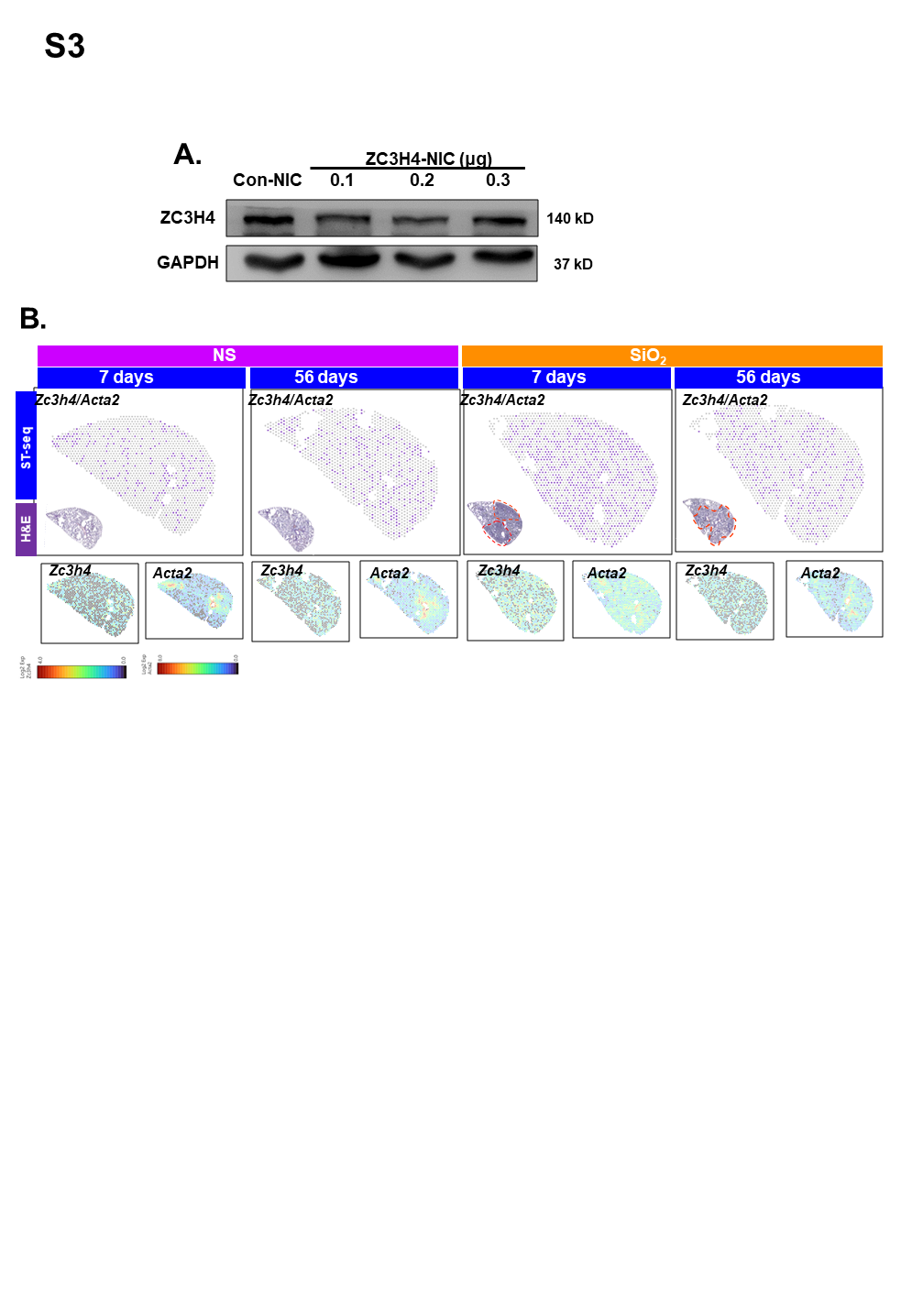


**Supplementary Figure S3.** (A) Representative western blot showing the transfection efficiency of ZC3H4-NICs in HPF-a cells. (B) Spatial transcriptomics showed the coexpression of *Zc3h4* and *Acta2*.

**Figure S4**


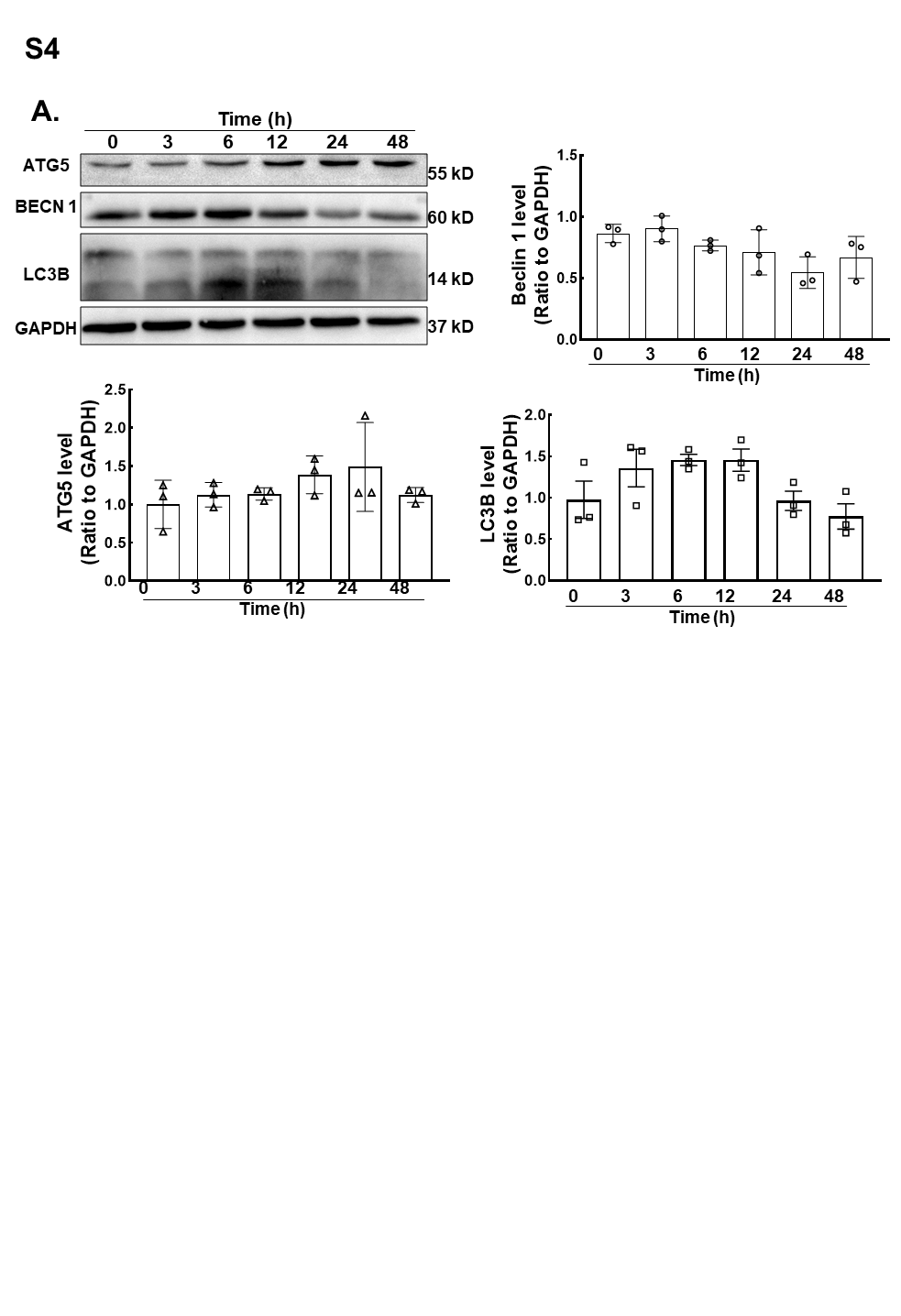


**Supplementary Figure S4.** The expression of autophagy-related proteins (ATG5, BECN1 and LC3B) after CM stimulation of HPF-a cells.

**Figure S5**


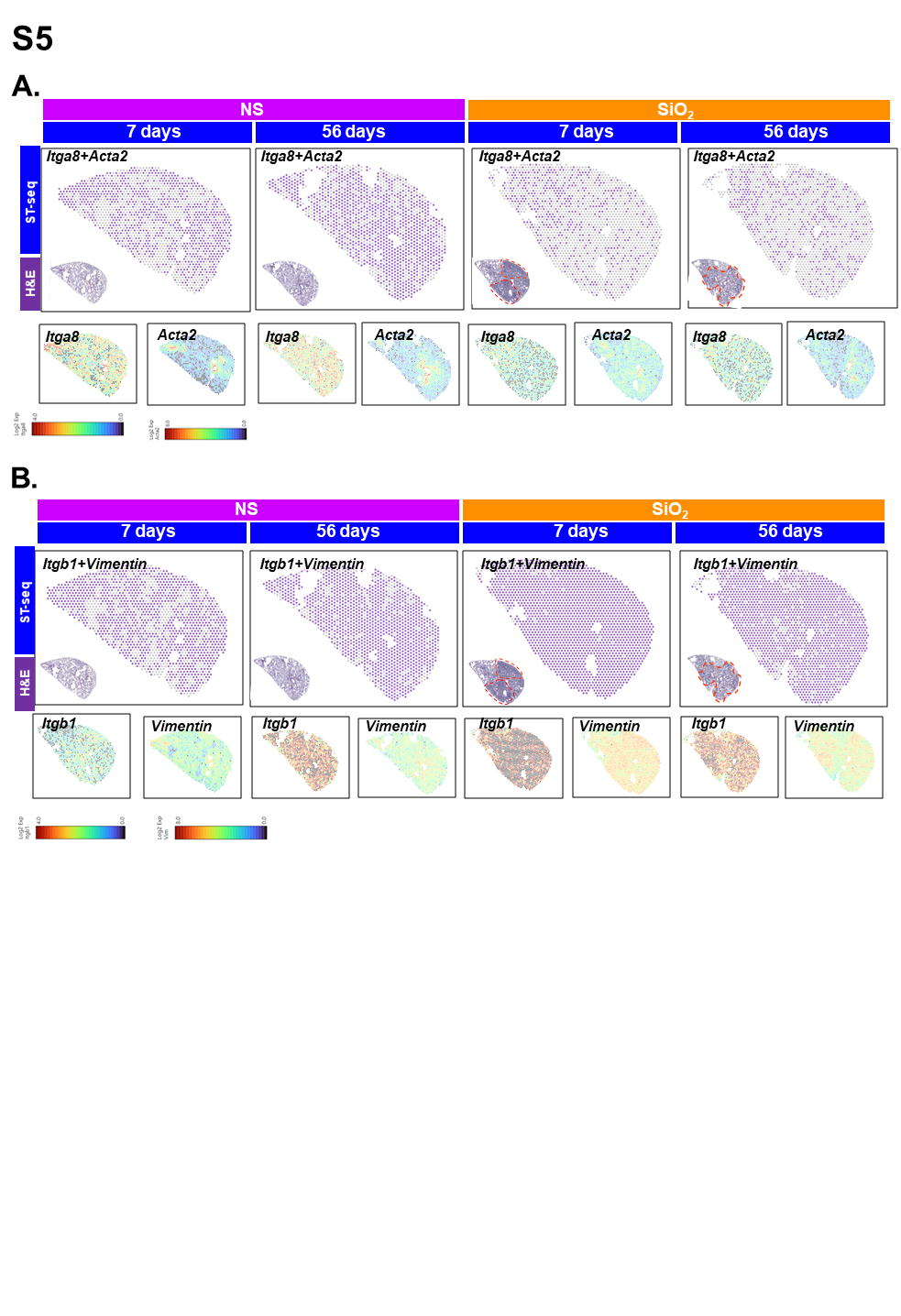


**Supplementary Figure S5.** (A) Spatial transcriptomics showed the coexpression of *Itga8* and *Acta2*. (B) Spatial transcriptomics showed the coexpression of *Itgb1* and *Vimentin*.
